# Oral Vancomycin for Prevention of Recurrent *Clostridioides difficile* Infection: A Randomized Clinical Trial

**DOI:** 10.1001/jamanetworkopen.2025.17834

**Published:** 2025-07-02

**Authors:** Julie A. Keating, Tinghui Xu, Mary Beth Graham, Mayur Ramesh, Sahil Khanna, Jonah Dixon, Ashley Kates, Kendra Haight, Jiwei Zhao, Christopher Saddler, Nasia Safdar

**Affiliations:** 1 Department of Medicine, School of Medicine and Public Health, University of Wisconsin–Madison; 2 William S. Middleton Memorial Veterans Hospital, Madison, Wisconsin; 3 Department of Biostatistics and Medical Informatics, School of Medicine and Public Health, University of Wisconsin–Madison; 4 Department of Statistics, University of Wisconsin–Madison; 5 UW Health, Madison, Wisconsin; 6 Division of Infectious Diseases, Department of Medicine, Medical College of Wisconsin, Milwaukee; 7 Division of Infectious Diseases, Henry Ford Hospital, Detroit, Michigan; 8 Division of Gastroenterology and Hepatology, Mayo Clinic, Rochester, Minnesota

## Abstract

**Importance:**

Systemic antibiotic use for patients with a non–*Clostridioides difficile* infection (CDI) is a major risk factor for recurrent CDI. Increasing use of oral vancomycin for secondary prophylaxis against recurrent CDI in this context has uncertain efficacy.

**Objective:**

To evaluate whether oral vancomycin prophylaxis compared with placebo is effective against recurrent CDI during and 8 weeks after the end of study treatment.

**Design, Setting, and Participants:**

This phase 2, placebo-controlled, double-blind randomized clinical trial was conducted in 4 large health systems across the upper Midwest US. Adults who had completed treatment for CDI within the past 180 days and were taking a systemic antibiotic for a non-CDI indication were enrolled between May 21, 2018, and March 30, 2023, and followed up for 8 weeks after the end of study treatment.

**Intervention:**

Participants were randomized 1:1 to 125 mg of oral vancomycin or placebo once daily during antibiotic use for a non-CDI plus 5 days following cessation of those antibiotics.

**Main Outcomes and Measures:**

The primary outcome was recurrent CDI incidence during treatment and the 8-week follow-up period. The secondary outcome was vancomycin-resistant *Enterococcus* carriage in stool.

**Results:**

Among 81 randomized participants (median age, 59 years [IQR, 50-67 years]), all were included in the primary as-randomized analysis (39 in the vancomycin group; 42 in the placebo group). Sixty patients (74.1%) completed 8-week follow-up and were included in the secondary as–completed treatment analysis (31 in the vancomycin group; 29 in the placebo group). Recurrent CDI occurred in 17 of 39 participants in the oral vancomycin group (43.6%) and 24 of 42 in the placebo group (57.1%; absolute difference in percentage, −13.5% [95% CI, −35.1% to 8.0%]). Adverse events occurred in 27 of 39 participants in the oral vancomycin group (69.2%) and 27 of 42 in the placebo group (64.3%). Vancomycin-resistant *Enterococcus* carriage was found in 15 of 30 patients in the oral vancomycin group (50.0%) and 6 of 25 in the placebo group (24.0%) (*P* = .048) 8 weeks after treatment.

**Conclusions and Relevance:**

In this randomized clinical trial, the incidence of recurrent CDI was lower (though did not reach significance) in participants taking oral vancomycin compared with those taking placebo. Because the study was underpowered, it was unable to reveal firm conclusions about the efficacy (or lack thereof) of vancomycin prophylaxis with respect to recurrent CDI.

**Trial Registration:**

ClinicalTrials.gov Identifier: NCT03462459

## Key Points

**Question:**

Is oral vancomycin prophylaxis effective for recurrent *Clostridioides difficile* infection (CDI) during antibiotic treatment for non-CDI indications?

**Findings:**

In this randomized clinical trial of 81 participants who had completed treatment for a CDI and were taking systemic antibiotics for a non-CDI indication, recurrent CDI occurred in 43.6% of participants in the oral vancomycin group vs 57.1% in the placebo group at 8 weeks. However, the study was underpowered and this difference did not reach statistical significance.

**Meaning:**

Other interventions should be investigated for their effectiveness in preventing CDI recurrence after non–CDI-indicated antibiotic therapy.

## Teaser

This randomized clinical trial examines whether prophylactic oral vancomycin vs placebo reduces recurrent *Clostridioides difficile* infection in adults receiving antibiotics for another indication.

## Introduction

*Clostridioides difficile* infection (CDI) is the most common cause of health care–associated diarrhea.^1,2^ Over one-third of patients who develop CDI will develop 1 or more recurrences; recurrence risk increases with each episode.^3,4^ The main risk factor for CDI is antibiotic use.^5^ Following therapy and resolution of initial CDI, recurrent CDI may be triggered when antibiotics are given for other indications.^2,6^ Prevention is essential to reduce the myriad medical, social, and psychological consequences of recurrent CDI.^7,8^ Interventions to prevent recurrent CDI during exposure to systemic antibiotics in a person with prior but not current CDI are needed in clinical practice.^9^

Oral vancomycin for prophylaxis against recurrent CDI has been used in clinical practice based on retrospective cohort studies showing possible favorable outcomes.^10,11^ However, their observational designs precluded assessing efficacy because of confounding, heterogeneity in data availability, inclusion criteria, study populations, and uncertainty regarding the magnitude of the effect.^12,13,14,15,16^ In a prospective trial in asymptomatic patients colonized with *C difficile*, oral vancomycin was effective in eliminating fecal *C difficile* during and immediately after treatment, but most participants began to shed *C difficile* again within approximately 3 weeks of completing oral vancomycin treatment.^17^

Whether oral vancomycin prophylaxis is effective against recurrent CDI remains unanswered. Furthermore, most studies have looked primarily at benefits of treatment without evaluating potential risks.^12,13,14,15,16,18^ This is a critical gap in the field, as oral vancomycin is not without risk (particularly, vancomycin-resistant *Enterococcus* [VRE] gut proliferation).^19,20,21,22^

We conducted a prospective randomized clinical trial to assess the effect of oral vancomycin prophylaxis on recurrent CDI incidence and VRE carriage of the gut in patients with a recent CDI who were taking systemic antibiotics for a non-CDI indication. This study aimed to evaluate whether a low dose of oral vancomycin administered during and immediately following non–CDI-indicated antibiotic treatment was effective in preventing recurrent CDI.

## Methods

### Trial Design

We conducted a phase 2, double-blind, placebo-controlled randomized clinical trial of adult patients with a recent CDI who were prescribed systemic antibiotics for non-CDI indications (NCT03462459). This was a multicenter study with participants enrolled at 4 large health systems: University of Wisconsin (UW) Health (Madison, Wisconsin) from May 21, 2018, through March 30, 2023; Medical College of Wisconsin (MCW; Milwaukee, Wisconsin) from February 1, 2019, through December 31, 2022; Henry Ford Hospital (Detroit, Michigan) from October 1, 2020, through December 31, 2021; and Mayo Clinic (Rochester, Minnesota) from November 1, 2021, through October 31, 2022. Three health systems (including UW Health and MCW) were initially included, with 1 dropping out without enrolling. Henry Ford Hospital was added in October 2020 and Mayo Clinic was added in November 2021 to increase enrollment. The trial protocol and statistical analysis plan are available in Supplement 1. The protocol was approved by the UW-Madison institutional review board, which served as the institutional review board of record for all study sites. All participants provided written informed consent via paper or electronic form. This study followed the Consolidated Standards of Reporting Trials (CONSORT) 2010 reporting guideline.

### Participants

Adults were eligible to participate if they had a documented diagnosis of 1 or more CDIs within the past 180 days, had completed CDI treatment, and were receiving non–CDI-indicated systemic antibiotics for 2 weeks or less. Participants could not have received more than 72 hours of systemic antibiotics at enrollment. Individuals were ineligible to participate if they had a contraindication to vancomycin use, were currently using oral vancomycin, or were using metronidazole or tetracycline monotherapy. Individuals were also ineligible if they had suspected CDI or other relevant gastrointestinal conditions, such as bacterial gastrointestinal infection, at enrollment; recent major gastrointestinal surgery; or history of total colectomy or bariatric surgery. A full list of inclusion and exclusion criteria is available in the trial protocol (Supplement 1).

Study coordinators (included K.H.) and investigators (physicians or advanced practice practitioners; included M.B.G., M.R., S.K., C.S., N.S.) reviewed inpatients’ and outpatients’ electronic medical records (EMRs) to determine their eligibility (positive *C difficile* test result within the past 180 days and a new antibiotic prescription for a non-CDI indication). Eligible individuals were approached and invited to attend an enrollment visit (visit 1), during which participants provided informed consent and underwent a clinical assessment to collect demographics, medical history, dietary information, baseline bowel movement measures (via the Bristol Stool Scale^23^), and current medications. Participants’ race and ethnicity data were required to be collected by the funding agency and were determined from EMR data. Racial groups participants could select were American Indian or Alaska Native, Asian, Black or African American, Native Hawaiian or Other Pacific Islander, White, and multiracial, and ethnic groups were Hispanic and non-Hispanic. Participants also provided a baseline stool sample or perirectal swab sample prior to beginning study treatment or placebo. Participants were then randomized to a study group and were provided the study intervention or placebo.

Patients had weekly telephone calls from their first study treatment dose through 8 weeks after completing treatment to monitor for adverse events and recurrent CDI. Patients had 2 visits after enrollment: visit 2 occurred within 1 week after the patient took their last study capsule, and visit 3 occurred 8 weeks after the patient took their last study capsule.

Recruitment and enrollment procedures were impacted by the COVID-19 pandemic, as nonessential clinical procedures, study enrollment, and in-person contact with patients and study participants were suspended in March 2020. The coordinating site received an exemption to continue the study after June 2020.

### Randomization

Participants were randomized in a 1:1 block scheme to the intervention (oral vancomycin) or placebo arm. A randomization schedule was created by the study biostatistician using random number generation software, and randomization was stratified by site. The randomization schedule was provided to site pharmacists, who distributed intervention or placebo materials directly to participants and/or clinical staff. Participants and study staff were blinded to study group assignment. Patients who had excess vancomycin or placebo doses after completing their treatment schedule were asked to return or dispose of the excess doses.

### Interventions

The intervention was 125 mg of oral vancomycin (Akorn). The placebo was 125 mg of lactose national formulary grade monohydrate powder encapsulated in empty size-1 gelatin (blue and white) capsules (both from Fagron). Sites purchased supplies and encapsulated study drug and/or placebo. Site investigational pharmacies could purchase a different generic from alternative sources if preferred materials were not available. Vancomycin and gelatin capsules that most closely resembled each other were selected.

Hospitalized patients received the study capsules from clinical staff, and treatment administration was scheduled during the stay. Outpatients self-administered capsules. Capsules were taken orally once per day for the duration of antibiotic use plus 5 days following cessation of non–CDI-indicated antibiotics. Participants recorded the dates of capsule ingestion on a medication diary.

### Outcomes

The primary objective was to evaluate the efficacy of oral vancomycin prophylaxis for recurrent CDI. The primary end point was incidence of recurrent CDI in participants using oral vancomycin compared with incidence in participants taking placebo during treatment and through 8 weeks following completion of treatment. Incidence of recurrent CDI was collected via weekly telephone calls with participants. Participants reporting 3 or more type-5 to type-7 stools (via the Bristol Stool Scale^23^) above their baseline bowel movements in a 24-hour period provided stool samples to the coordinating site laboratory for 2-step testing to confirm recurrent CDI. Stool samples were cultured for *C difficile*, with toxigenic polymerase chain reaction confirmation for positive culture results. A combined *C difficile* toxin A and B enzyme-linked immunoassay (ELISA) (TECHLAB Inc) conducted by the coordinating site laboratory followed by a cytotoxin cell culture assay (if ELISA results were negative) at ARUP Laboratories was used to identify toxigenic *C difficile* for the recurrent CDI outcome.

The secondary objective was comparing gut VRE carriage in patients taking oral vancomycin vs placebo. Patients self-collected stool samples (and/or perirectal swab samples if stool could not be produced) at enrollment, visit 2, and visit 3 and shipped them to the coordinating site laboratory. The presence of VRE was identified through broth enrichment following culture onto selective agar.

### Sample Size

Power and sample size were based on the primary outcome (recurrent CDI incidence). Previous research showed an absolute decrease of 22% in the proportion of patients with CDI recurrence (*h* = 0.66) between those receiving oral vancomycin and those receiving placebo.^11^ We estimated that using a 1:1 random allocation ratio with 80% power and 2-tailed *P* < .05, a sample size between 84 and 144 total participants would be needed depending on the level of attrition. We proposed a total sample size of 150 participants, anticipating no more than a 20% attrition rate, to detect effects as low as *h* = 0.57, or a 20.5% decrease in recurrence.

### Statistical Analysis

Statistical analyses are primarily reported for the population randomized in the study (as-randomized group). The secondary statistical analyses are reported for the population that completed all 3 visits (as–completed treatment group). Proportional tests were conducted to compare the episodes of CDI recurrence in the 8 weeks following the completion of the study intervention in patients receiving vancomycin vs patients receiving placebo using the χ^2^ test. Log-rank tests were conducted to compare the proportions without recurrence within the 8-week period between patients receiving vancomycin and those receiving placebo with the Kaplan-Meier method. A nonparametric Wilcoxon rank sum test was used to compare distributions of the number of days to the first recurrence of CDI after starting oral vancomycin or placebo. The χ^2^ test was also conducted to compare the proportions of patients who had positive VRE results. The significance level was set to 2-sided *P* ≤ .05. All statistical analyses were conducted in R, version 4.4.0 (R Project for Statistical Computing).^24^

## Results

### Participants

**Figure 1.**
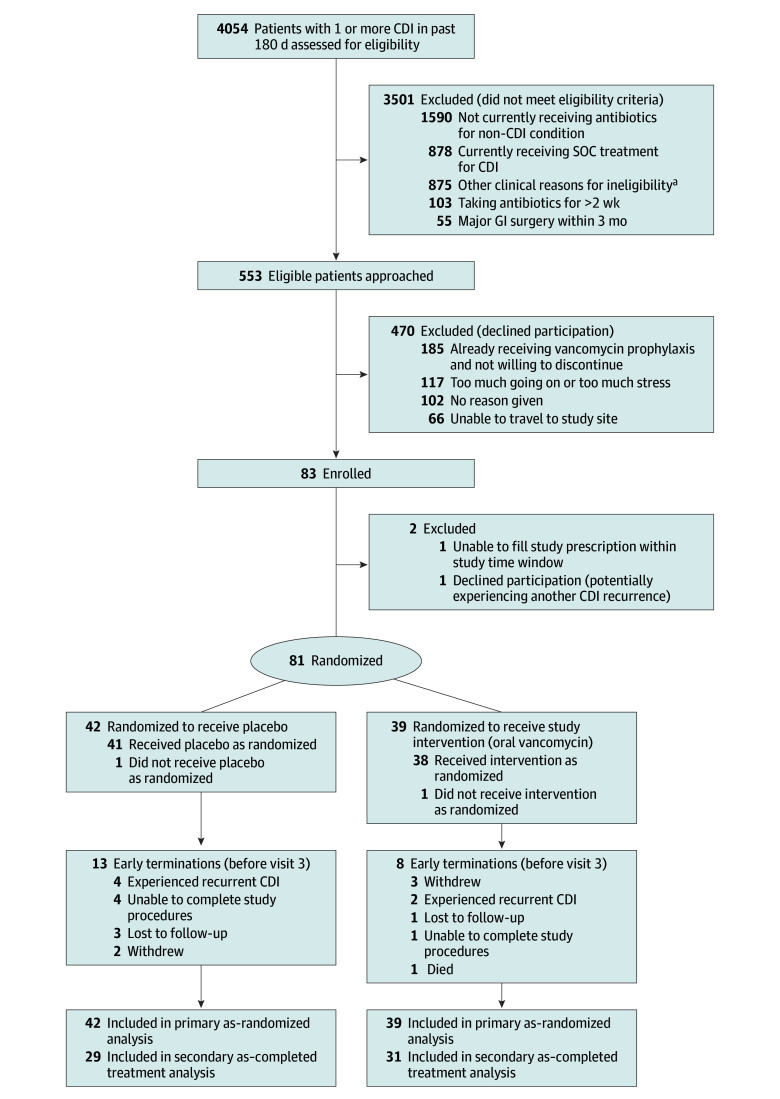
Participant Selection and Flow in the Trial of Oral Vancomycin for the Prevention of Recurrent *Clostridioides difficile* Infection (CDI) GI indicates gastrointestinal; SOC, standard of care. ^a^Other clinical reasons for exclusion included current diarrhea; pregnancy or lactation; life expectancy under 6 months; died after screening but before being approached by study team; other known (non-CDI) bacterial infection, toxic megacolon, or small bowel ileus; known to be colonized with *C difficile*; or other complex medical history or inappropriate for study per clinician (eg, total colectomy or bariatric surgery, allergy to oral vancomycin, or inability to swallow capsules).

Between May 21, 2018, and March 30, 2023, 4054 individuals were assessed for eligibility via EMR, and 553 eligible individuals were approached. Of those, 470 declined participation, including 185 (39.4%) who were already taking oral vancomycin and did not wish to discontinue (156 of the 185 [84.3%] were at a single site). A total of 83 individuals enrolled, and 81 were randomized (39 to oral vancomycin and 42 to placebo) (Figure 1). Table 1 displays baseline characteristics including demographics and medical history within each treatment group (oral vancomycin or placebo). Median age of the participants was 59 years (IQR, 50-67 years); 49 (60.5%) were female, and 32 (39.5%) were male. One participant (1.2%) was American Indian or Alaska Native, 1 (1.2%) was Asian, 9 (11.1%) were Black or African American, none were Native Hawaiian or Other Pacific Islander, 68 (84.0%) were White, none were multiracial, and 2 (2.5%) had unknown race. Of 80 participants with ethnicity data, 2 (2.5%) were Hispanic and 78 (97.5%) were non-Hispanic. Demographic factors and baseline comorbid conditions were evenly distributed across study groups.

**Table 1.** Baseline Participant Demographics and Medical History

Characteristic	Patients^a^	
	Oral vancomycin (n = 39)	Placebo (n = 42)
Age, median (IQR), y	58 (49-67)	59 (50-66)
Sex		
Female	24 (61.5)	25 (59.5)
Male	15 (38.5)	17 (40.5)
Race		
American Indian or Alaskan Native	0	1 (2.4)
Asian	1 (2.6)	0
Black or African American	3 (7.7)	6 (14.3)
Native Hawaiian or Other Pacific Islander	0	0
White	34 (87.2)	34 (81.0)
Multiracial	0	0
Unknown	1 (2.6)	1 (2.4)
Ethnicity		
Hispanic	1/39 (2.6)	1/41 (2.4)
Non-Hispanic	38/39 (97.4)	40/41 (97.6)
Smoking status		
Have not smoked within 6 mo	32/33 (97.0)	35/38 (92.1)
Smoked within 6 mo	1/33 (3.0)	3/38 (7.9)
Fecal microbiota transplant history		
Yes	1/39 (2.6)	0
No	38/39 (97.4)	41/41 (100)
Medical history		
Head, ears, nose, throat	26 (66.7)	29 (61.9)
Respiratory	28 (71.8)	22 (52.4)
Cardiovascular	32 (82.1)	30 (71.4)
Gastrointestinal	33 (84.6)	36 (85.7)
Genitourinary	34 (87.2)	35 (83.3)
Musculoskeletal	28 (71.8)	35 (83.3)
Neurologic	16 (41.0)	21 (50.0)
Endocrine or metabolic	30 (76.9)	32 (76.2)
Lymphatic	17 (43.6)	10 (23.8)
Dermatologic	17 (43.6)	13 (31.0)
Psychiatric	23 (59.0)	24 (57.1)
Other	22 (56.4)	26 (61.9)
Major surgery	2 (5.1)	4 (9.5)
Unknown or not reported	0	2 (4.7)

^a^ Data are presented as number or number/total number (percentage) of participants unless otherwise indicated.

Because of COVID-19 pandemic impacts and lack of funding for project extensions, enrollment ended at 81 participants. Overall, 55 (67.9%) were enrolled at UW Health, 18 (22.2%) at MCW, 6 (7.4%) at Henry Ford Hospital, and 2 (2.5%) at Mayo Clinic. A total of 60 participants (74.1%), with 29 in the placebo group (69.0%) and 31 in the oral vancomycin group (79.5%), completed all study activities through visit 3 and were included in the secondary as–completed treatment analysis (eTable 1 in Supplement 2).

### Primary Outcome

**Figure 2.**
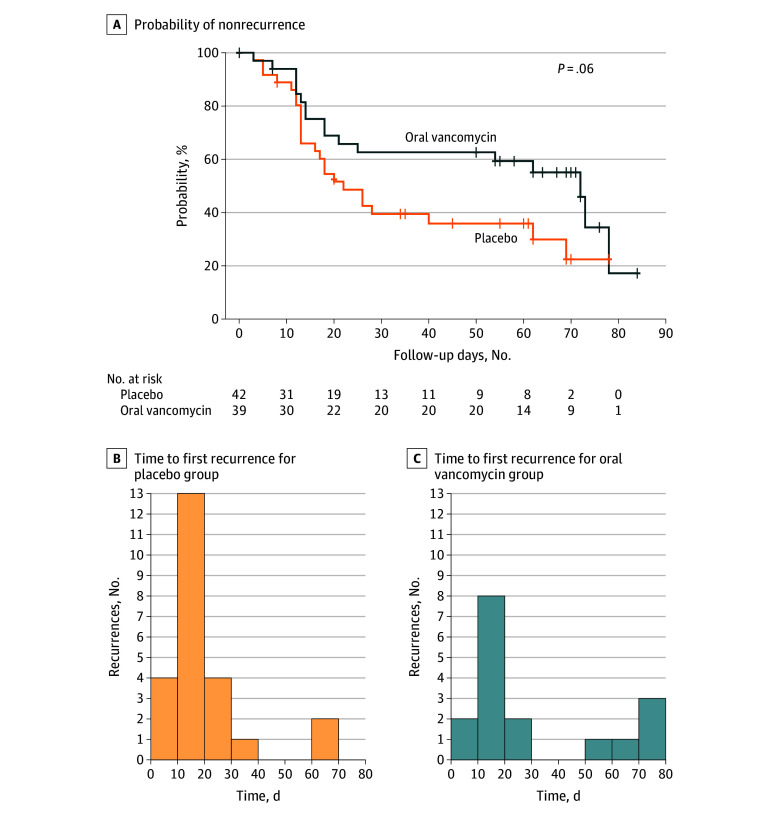
Estimated Nonrecurrence Probability Over Time Between Treatment Groups for the As-Randomized Population and Distribution of Number of Days to First Recurrence of *Clostridioides difficile* Infection After Beginning Oral Vancomycin or Placebo Day 0 corresponds to the first day of oral vancomycin or placebo use as part of this study.

Eight weeks after completing treatment, 17 of 39 patients in the oral vancomycin group (43.6%) and 24 of 42 in the placebo group (57.1%) experienced a CDI recurrence (absolute difference in percentage, −13.5%; 95% CI, −35.1% to 8.0%; *P* = .22) (Table 2). There was no statistically significant difference in nonrecurrence probability over time (from start of oral vancomycin or placebo treatment) between treatment groups (Figure 2A). We also did not find a difference in the distribution of the number of days to first recurrence of CDI between treatment groups (Figure 2B and C). Similar results were observed in the as–completed treatment analysis (eAppendixes 1 and 2, eTable 1, and eFigure 1 in Supplement 2). There was a slight difference between curves when comparing CDI recurrence between study sites (eAppendix 2, eTable 2, and eFigure 2 in Supplement 2). There was no difference in recurrent CDI outcomes when evaluating subgroups at particularly high risk of recurrent CDI (use of high-risk antibiotics, having an immunocompromised condition, or age ≥65 years) (eAppendixes 3-5, eTables 3-5, and eFigures 3-5 in Supplement 2).^25,26^

**Table 2.** As-Randomized Analysis of Primary and Secondary Outcomes^a^

Outcome	Patients, No. (%)		Absolute difference in percentage, % (95% CI)	*P* value
	Oral vancomycin	Placebo		
Primary outcome				
CDI recurrence within 8 wk	17/39 (43.6)	24/42 (57.1)	−13.5 (−35.1 to 8.0)	.22
Secondary outcome^b^				
VRE carriage at visit 1	20/37 (54.1)	27/40 (67.5)	−13.4 (−35.1 to 8.2)	.23
VRE carriage at visit 3	15/30 (50.0)	6/25 (24.0)	26.0 (1.5 to 50.5)	.048

Abbreviations: CDI, *Clostridioides difficile* infection; VRE, vancomycin-resistant enterococcus.

^a^ The primary and secondary outcomes were analyzed using a χ^2^ test without continuity correction.

^b^ Stool samples to measure VRE carriage were collected within 14 days of 8 weeks following the end of treatment. VRE carriage results were missing from 9 patients in the oral vancomycin group (23.1%) and 17 patients in the placebo group (40.5%) at visit 3.

### Secondary Outcome

There was no difference in VRE carriage between treatment groups at enrollment (visit 1) (Table 2). Participants taking oral vancomycin had marginally more VRE carriage 8 weeks after completing treatment (visit 3) (15 of 30 [50.0%]) than participants taking placebo (6 of 25 [24.0%]) (absolute difference in percentage, 26.0%; 95% CI, 1.5%-50.5%; *P* = .048). However, in the placebo group, there was a significant reduction in participants with VRE carriage between visit 1 (27 of 40 [67.5%]) and visit 3 (6 of 25 [24.0%]) (absolute difference in percentage, 43.5%; 95% CI, 21.3%-65.7%; *P* < .001). There was no difference in VRE carriage between visit 1 (20 of 37 [54.1%]) and visit 3 (15 of 30 [50.0%]) in the oral vancomycin group (absolute difference in percentage, 4.1%; 95% CI, −20.0% to 28.1%; *P* = .74).

### Adverse Events

In the as-randomized population, 27 participants in the oral vancomycin group (69.2%) and 27 participants in the placebo group (64.3%) reported 1 or more adverse events (Table 3). Of all 88 adverse events, 85 (96.6%) were deemed to be unrelated or unlikely to be related to the study treatment. The most common adverse event was gastrointestinal disorders, reported in 20 participants taking oral vancomycin (51.3%) and 20 participants taking placebo (47.6%).

**Table 3.** Adverse Events During the 8 Weeks Following Treatment

Adverse event	Patients, No. (%)	
	Vancomycin arm (n = 39)	Placebo arm (n = 42)
Any	27 (69.2)	27 (64.3)
Blood and lymphatic system disorders	1 (2.6)	0
Cardiac system disorders	2 (5.1)	0
Ear and labyrinth disorders	0	1 (2.4)
Endocrine disorders	1 (3.3)	0
Gastrointestinal disorders	20 (51.3)	20 (47.6)
General disorders and administration site conditions	3 (7.7)	3 (7.1)
Infections and infestations	8 (20.5)	6 (14.3)
Injury, poisoning, and procedural complications	0	2 (4.8)
Investigations	0	1 (2.4)
Metabolism and nutrition disorders	1 (2.6)	1 (2.4)
Musculoskeletal and connective tissue disorders	4 (10.3)	1 (2.4)
Nervous system disorders	1 (2.6)	2 (4.8)
Psychiatric disorders	1 (2.6)	1 (2.4)
Kidney and urinary disorders	4 (10.3)	0
Reproductive system and breast disorders	0	1 (2.4)
Respiratory, thoracic, and mediastinal disorders	1 (2.6)	1 (2.4)
Skin and subcutaneous tissue disorders	2 (5.1)	2 (4.8)
Surgical and medical procedures	2 (5.1)	2 (4.8)
Vascular disorders	0	4 (9.5)

## Discussion

In this double-blind, placebo-controlled randomized clinical trial, patients who had CDI in the previous 180 days who took 125 mg of prophylactic oral vancomycin during and for 5 days after a course of non–CDI-indicated antibiotic therapy had lower rates of recurrent CDI than patients taking a placebo; however, this study was underpowered to find a statistically significant difference. There were high rates of recurrent CDI in both the treatment (43.6%) and the placebo (57.1%) groups. However, the study population was at particularly high risk for recurrent CDI given that all participants had a recent CDI and used systemic antibiotics.^26,27^ The median age of participants was 59 years, and older age is also a risk factor for recurrent CDI.^26^

Previous prospective randomized clinical trials have had mixed findings on the effectiveness of oral vancomycin prophylaxis. A trial in asymptomatic patients with *C difficile* colonization found that oral vancomycin was temporarily effective in reducing *C difficile* shedding during and immediately after treatment, but most patients receiving oral vancomycin returned to shedding *C difficile* within approximately 3 weeks of completing treatment.^17^ Another randomized trial of oral vancomycin to prevent initial CDI in high-risk patients receiving systemic antibiotics found a significant reduction in health care facility–onset CDI in patients receiving oral vancomycin prophylaxis compared with no prophylaxis.^18^ However, the follow-up period was limited. We had a longer follow-up period of 8 weeks to measure recurrent CDI incidence after completion of treatment.

In the absence of clear evidence, use of oral vancomycin prophylaxis has remained an unresolved issue among leading health organizations. Oral vancomycin prophylaxis during non–CDI-indicated systemic antibiotic therapy has been conditionally recommended for patients at high risk of CDI recurrence by the American College of Gastroenterology (ACG), which notes that this recommendation is supported by low-quality evidence and that there is a need for prospective clinical trials.^28^ The Infectious Diseases Society of America found insufficient evidence to make a recommendation in its 2017 clinical practice guidelines and did not address the question in its 2021 update.^29,30^ The European Society of Clinical Microbiology and Infectious Diseases 2021 update did not recommend routine prophylaxis during antibiotic treatment but suggested prophylaxis may be appropriate in select patients with recurrent CDI after antibiotic use and with careful consideration of risks and benefits.^31^

Our clinical trial used the ACG dosage and treatment duration for prophylactic oral vancomycin (125 mg of oral vancomycin once per day for the duration of non–CDI-indicated antibiotic therapy plus 5 days after the course of antibiotics was completed). Previous studies have evaluated different dosages and durations of oral vancomycin treatment.^17,18^ We selected a low dosage to reduce the risk of gut dysbiosis. A meta-analysis of oral vancomycin prophylaxis in a variety of patient populations found recurrent CDI to be less likely in patients taking oral vancomycin.^32^ In addition, recent meta-analyses found the lower dosage and longer duration that we used in our study to be associated with protective effects against CDI.^32,33^ However, nearly all prior studies on this topic have been retrospective.^9,32^ A strength of our study was its design as a placebo-controlled, double-blind randomized clinical trial, allowing for prospective analyses of the effectiveness of oral vancomycin in reducing recurrent CDI outcomes compared with placebo. Because our study was underpowered, we were not able to confirm these previous findings^10,11,32,33^; we did not find a significant reduction in recurrent CDI in patients prospectively assigned to use of oral vancomycin compared with those receiving placebo.

Vancomycin use has been associated with VRE colonization.^34^ While other studies have not found an increase in VRE infections after oral vancomycin treatment,^32^ our study evaluated carriage of VRE in the gut, a risk factor for future VRE infection.^34^ We found that in patients taking placebo, there was a significant reduction in the proportion with VRE carriage from visit 1 (27 of 40 [67.5%]) to visit 3 (6 of 25 [24.0%]) (*P* < .001), but VRE carriage did not change in patients taking oral vancomycin.

Prior to and during our study, clinical oral vancomycin prophylaxis use at participating sites was often based on institutional and practice preferences rather than clinical trial evidence to show benefit. Many patients were prescribed oral vancomycin prophylaxis prior to being identified for possible enrollment in this study. During recruitment, we noted many participants who did not wish to participate because they were already taking oral vancomycin prophylaxis and did not wish to discontinue use due to the possibility of being randomized to the placebo group. Of the 470 eligible patients who were approached but declined participation, 185 (39.4%) cited this concern. Of these 185 patients, 156 (84.3%) were located at 1 site. Given the difference in protocols for preventing recurrent CDI across different sites and the impact of this on potential patient populations, maintaining equipoise will be a consideration in any future studies.

### Limitations

This study has limitations. We did not reach our target enrollment of 150 participants, ultimately randomizing 81 participants. While we have reported the trends and findings in this population, the study was underpowered, which precludes drawing conclusions from findings.

Our study also did not evaluate broader effects on the gut microbiome that may be impacted by systemic antibiotic and oral vancomycin use. Vancomycin alters the gut microbiome, including reducing baseline commensals, which may allow *C difficile* to colonize and establish.^35,36^ Additional research on patient populations, dosages, and dosing schedules may be needed to determine whether oral vancomycin may provide a greater protective effect within specific parameters while maintaining a low risk profile for patients.

## Conclusions

In this randomized clinical trial, among patients with a recent CDI and taking a subsequent non–CDI-indicated antibiotic course, patients taking oral vancomycin had reduced rates of recurrent CDI compared with patients taking a placebo. However, our study was underpowered to identify a statistically significant difference. There was a marginal but significant difference in VRE carriage in stool samples at 8 weeks after treatment between patients taking oral vancomycin and patients taking a placebo; however, participants taking placebo had a decrease in VRE carriage after completing treatment that was not observed in participants taking oral vancomycin. While this study was underpowered due to enrollment challenges, these findings suggest that other interventions should be investigated for their effectiveness in preventing CDI recurrence after non–CDI-indicated antibiotic therapy.

## Notes

Supplement 1.
Trial Protocol

Supplement 2. **eAppendix 1.** Secondary As-Completed-Treatment Analyses Results **eTable 1.** As-Completed-Treatment Analysis of Primary and Secondary Outcomes **eFigure 1.** Estimated Nonrecurrence Probability Over Time Between Treatment Groups for the As-Completed-Treatment Population **eAppendix 2.** Comparison of Primary Outcome, Stratified by Study Sites **eFigure 2.** Estimated Nonrecurrence Probability Over Time Between Treatment Groups for the As-Randomized Population, Stratified by Study Sites **eTable 2.** Comparison of Recurrence Rates Between Intervention Group and Placebo Group at 10-Day Intervals After the Intervention, Stratified by Study Sites **eAppendix 3.** Primary and Secondary Outcomes in Participants Taking Higher-Risk Antibiotics **eTable 3.** Primary and Secondary Outcomes in Participants Taking High-Risk Antibiotics **eFigure 3.** Estimated Nonrecurrence Probability Over Time Between Treatment Groups for the Randomized Participants Taking High-Risk Antibiotics **eAppendix 4.** Primary and Secondary Outcomes in Immunocompromised Participants **eTable 4.** Primary and Secondary Outcomes in Immunocompromised Participants **eFigure 4.** Estimated Nonrecurrence Probability Over Time Between Treatment Groups for the Randomized Participants With an Immunocompromising Condition **eAppendix 5.** Primary and Secondary Outcomes in Participants Aged 65 Years or Older **eTable 5.** Primary and Secondary Outcomes in Participants Aged 65 Years or Older **eFigure 5.** Estimated Nonrecurrence Probability Over Time Between Treatment Groups for the Randomized Participants Aged 65 Years or Older

Supplement 3.
Data Sharing Statement
