# Supplementary material for: Oral Vancomycin for Prevention of Recurrent Clostridioides difficile Infection: A Randomized Clinical Trial
Source: JAMA Netw Open. 2025 Jul 2;8(7):e2517834. doi: 10.1001/jamanetworkopen.2025.17834 (PMC12223870; doi:10.1001/jamanetworkopen.2025.17834)
Supplement: Supplement 1. — Trial Protocol [file jamanetwopen-e2517834-s001.pdf]

A Randomized Controlled Trial of Oral Vancomycin for Preventing Recurrent *Clostridium difficile* Infection

Full title: A phase II randomized, double-blinded, placebo-controlled, multicenter trial evaluating the efficacy of oral vancomycin prophylaxis for prevention of recurrent *Clostridium difficile* Infection

Principal Investigators: Nasia Safdar (Lead PI)
Mary Beth Graham (Site PI)
Mayur Ramesh (Site PI)
Sahil Khanna (Site PI)

Sites: University of Wisconsin
Medical College of Wisconsin
Henry Ford Hospital System
Mayo Clinic - Rochester

This document is confidential.
No part of it may be transmitted, reproduced, published, or used by other persons without prior written authorization from the Sponsor or Principal Investigator.

PROTOCOL VERSION and AMENDMENTS

Protocol Version	Date	Change Initiated (Initials)	Brief description of protocol modification/actions requested, if any
Version 1	12/27/16	NS	
Version 2.1	02/09/2018	KH	
Version 2.2	03/19/2018	KH	
Version 3	9/4/2018	KH	
Version 3.1	11/01/2018	KH	

Version 4.0	1/7/2019	IG	1. Increased eligibility timeline from 90 to 180 days from initial CDI<br>2. Added passive chart review: Visit 3 to 6-month follow-up phone call<br>3. Removed exclusion for FMT<br>4. Added waiver of consent option for initial stool sample<br>5. PE/vitals can be extracted from chart if within 2 weeks of enrollment<br>6. Added option for blood pregnancy test (or negative pregnancy test in chart if performed within seven days of enrollment) in addition to existing urine test. Added risk of blood draw to risk section.<br>7. Clarified it is acceptable for patients to be taking antibiotics administered via inhalation concurrently with study participation (not exclusionary)
Version 4.1	5/8/2019	IG	1. Now including patients with antibiotic course >14 days if prescribed due to logistics (e.g. scheduling) (inclusion #5 footnote).<br>2. Clarified diarrhea at time of enrollment is permissible if explainable by reasons other than CDI or CDI test is negative within 72 hours of enrollment (exclusion #13).<br>3. Updated AE reporting for eCRF recording to omit events that are unrelated to study participation if grade 1 or 2; grade 3 or higher will be recorded regardless of relatedness.<br>4. Added line explaining toxin detection cannot be completed on perirectal swabs.<br>5. Clarified dosing for patients who complete non-CDI antibiotics prior to first dose of study vancomycin/placebo.
Version 4.2	7/8/2019	KH	1. Clarified language regarding concurrent enrollment when a patient is receiving investigational drugs<br>2. Changed study vancomycin/placebo duration to be able to be lengthened if a patient's non-CDI antibiotic is lengthened while the patient is still receiving study product.
Version 5	10Oct2019	KH	1. Removed references to Indiana University<br>2. Increased patient stipend<br>3. Added text message option to recruit patients<br>4. Allowed patients lacking capacity to join the study<br>5. Removed requirement for patients to be seen in person to enroll<br>6. Reworded concurrent acceptable long-term antibiotic therapy<br>7. Removed physical exam requirement<br>8. Clarified only bacterial Gi infections that cause diarrhea should be excluded
Version 5.1	18Dec2019	JP	1. Added Henry Ford Hospital System as a local site<br>2. Corrected Patient total stipend
Version 6	18Nov2020	JP	1. When possible, distribute 5 days of study medication to subjects who have completed non-CDI dosing prior to enrolling, and those receiving one dose of non-CDI antibiotic.
Version 7	10JUN2021	JP	1. Added Mayo Clinic- Rochester as a local site
Version 8	26OCT2021	JP	1. Update MCW PI, update protocol contact information

PROTOCOL SIGNATURE PAGE

I have read the foregoing protocol and agree that it contains all necessary details for carrying out this study. I will conduct the study in accordance with the design and specific provisions outlined herein; deviations from the protocol are acceptable only with a mutually agreed upon protocol amendment.

I will provide copies of the protocol and all pertinent information to all individuals responsible to me who assist in the conduct of this study. I will discuss this material with them to ensure they are fully informed regarding the study drug or device and the conduct of the study.

I will use the informed consent form approved by the IRB and will fulfill all responsibilities for submitting pertinent information to the Institutional Review Board or Ethics Committee responsible for this study.

I also agree to report all information or data in accordance with the protocol and, in particular, I agree to report any serious adverse experiences as defined in Section 10 of this protocol.

I further agree that the funding agency and IRB has access to any source documents from which case report form information may have been generated.

I also agree to handle all clinical supplies (including study drug or device) and collect and handle all clinical specimens in accordance with the protocol.

The below signed confirm herewith to have read and understood this trial protocol and/or amendment and appendices; furthermore, to accomplish this study in accordance to the protocol and Good Clinical Practice guidelines, as well as local regulations; and to accept respective revisions conducted by regulatory authorities.

PRINTED OR TYPED NAME(S)	SIGNATURE	DATE
______________________	______________________	______________________
Principal Investigator(s)		
______________________	______________________	______________________

STATEMENT OF COMPLIANCE

The study will be carried out in accordance with Good Clinical Practices (GCP) as required by the following:

- United States Code of Federal Regulations (CFR) applicable to clinical studies (45 CFR Part 46; 21 CFR Part 50, 21 CFR Part 54, 21 CFR Part 56, and 21 CFR Part 312);
- International Conference on Harmonization (ICH) E6; 62 Federal Register 25691 (1997);

Compliance with these standards provides public assurance that the rights, safety and well-being of study subjects are protected, consistent with the principles that have their origin in the Declaration of Helsinki.

All key personnel (all individuals responsible for the design and conduct of this study) have completed Human Subjects Protection Training.

I agree to ensure that all staff members involved in the conduct of this study are informed about their obligations in meeting the above commitments.

Principal Investigator: ______________________________
Print/Type Name

Signed: _______________________________________

**Funding Sponsor:**	Agency for Healthcare Research and Quality(AHRQ)
**Study Product:**	Oral Vancomycin (Vancomycin)
**IND/IDE Number:**	N/A
**Participating sites:**	University of Wisconsin<br>Medical College of Wisconsin<br>Henry Ford Hospital System<br>Mayo Clinic - Rochester

List of Abbreviations

**AE**	Adverse event
**AHRQ**	Agency for Healthcare Research and Quality
**CDI**	*Clostridium difficile* Infection
**CFR**	Code of Federal regulations
**CRF**	Case Report Form
**CRO**	Contract Research Organization
**DCC**	Data Coordinating Center
**DMC**	Data Monitoring Committee
**eCRF**	Electronic Case Report Forms
**FDA**	Food and Drug Administration
**GCP**	Good Clinical Practice
**HIPAA**	Health Insurance Portability and Accountability Act
**ICF**	Informed Consent Form
**ICH E6**	International Conference on Harmonisation Guidance for Industry, Good Clinical Practice: Consolidated Guidance
**ICMJE**	International Committee of Medical Journal Editors
**ICTR**	Institute for Clinical and Translational Research
**IND**	Investigational New Drug Application
**IRB**	Investigational Review Board
**MOP**	Manual of Procedures
**N**	Number (typically refers to subjects)
**OHRP**	Office for Human Research Protections
**PHI**	Protected Health Information
**PI**	Principal Investigator
**QA**	Quality Assurance
**QC**	Quality Control
**REDCap**	Research Electronic Data Capture
**SAE**	Serious Adverse Event
**SMS**	Study Monitoring Service
**VRE**	Vancomycin Resistant Enterococcus

Study Summary

Title	A Phase II randomized, double-blinded, placebo-controlled, multicenter trial evaluating the efficacy of oral vancomycin prophylaxis for prevention of recurrent *Clostridium difficile* Infection.
Short Title and Precis	A Randomized Controlled Trial of Oral Vancomycin for Preventing Recurrent *Clostridium Difficile* Infection
ClinicalTrials.gov number	NCT03462459
Phase	Clinical study phase II
Methodology	Double blind, randomized, placebo control design
Study Duration	5 years (3 and a half years for recruitment, remainder for follow-up and analysis)
Study Center(s)	Multi-center study at 3 tertiary academic medical centers
Objectives	Primary objective:<br>To evaluate the efficacy of oral vancomycin prophylaxis for preventing recurrent *Clostridium difficile* infection (CDI) in patients who have experienced an initial episode of CDI in the last 180 days and are receiving antibiotics for a non-CDI indication.<br>Secondary objectives:<br>-To compare the diversity and function of the gut microbiota between the treatment and placebo group.<br>-To determine gut colonization by vancomycin-resistant enterococcus (VRE) in patients receiving intervention vs placebo.<br>-To determine if *C. difficile* positivity on any stool sample is a predictor for CDI recurrence.
Number of Subjects	150 who complete treatment and follow-up or are removed due to CDI recurrence (1:1 allocation ratio - 75 in the treatment group and 75 in the placebo group). Approximately 50 participants will be enrolled from each of the 3 sites.
Diagnosis	Recurrent CDI
Main Inclusion Criteria	Male or non-pregnant female patients age 18 years or older, able to provide a signed and dated informed consent; Patients who have experienced an initial CDI episode in the last 180 days and are receiving antibiotics for a non CDI indication
Main Exclusion Criteria	No prior history of CDI; inability (e.g. dysphagia) to or unwilling to swallow capsules; allergy to vancomycin
Study Product, Dose, Route, Regimen	Oral vancomycin 125 milligrams-once daily
FDA status of product	An IND is not required based on the following criteria: oral vancomycin in 125mg capsules is FDA approved; this study is not intended to provide support for any vancomycin labeling or advertising changes, or promote its commercialization; and the dosing and route of administration used in this protocol does not increase any risks associated with vancomycin.
Duration of administration	Oral vancomycin or placebo will be given for the duration of antibiotic therapy plus 5 days
Reference therapy	Placebo
Statistical Methodology	Basic proportional difference tests to compare CDI recurrence between the groups. Intention to treat analysis using Kaplan-Meier method.<br>Multivariate Cox proportional hazard regression will be used to determine predictors of CDI recurrence.<br>Age adjusted Cox proportional analysis will be used to determine time to recurrence with vancomycin treatment.

This page is intentionally left blank.

Contents

Study Summary 7
1. Key Roles 12
2. Background and Introduction 13
2.1 Background and Scientific Rationale 13
2.2 Hypothesis 15
2.3 Study Agent 16
2.4 Summary of Clinical Data 16
2.5 Dose Rationale 16
2.6 Potential Risk and Benefits to Subjects 16
2.6.1 Known Potential Risks 16
2.6.2 Protection Against Risks 17
2.6.3 Potential Benefits to the Subjects 17
3 Study Objectives and Purpose 17
4 Study Design and Endpoints 18
4.1 General Design 18
4.1.1 Primary Study Endpoint: 21
4.1.2 Secondary Study Endpoints: 21
4.1.3 Primary Safety Endpoint: 21
5 Study Subjects – Enrollment and Withdrawal 22
5.1 Subject Population 22
5.2 Subject Screening for Recruitment 22
5.2.1 Inclusion Criteria 22
5.2.2 Exclusion Criteria 23
5.3 Subject Identification 23
5.3.1 Recruitment and Retention Strategies 23
5.4 Vulnerable Populations 24
5.5 Subject Capacity 25
5.5.1 Subject Comprehension 25
5.6 Informed Consent 25
5.6.1 Process of Consent 25
5.6.2 Consent Form 26
5.6.3 HIPAA 26
5.6.4 Revoking Consent 26
5.6.5 Costs to the Subject 26
5.6.6 Payment for Participation 26
5.7 Early Withdrawal of Subjects 27
5.7.1 Premature termination of study 27

5.7.2 When and How to Withdraw Subjects 27
5.7.3 Data Collection and Follow-up for Withdrawn Subjects 27
6 Study Agent 28
6.1 Description and Formulation 28
6.2 Packaging 28
6.3 Preparation, Administration and Storage of Study Drug 28
6.4 Route of Administration 28
6.5 Starting Dose and Dose Escalation Schedule 28
6.6 Dose Adjustments/Modifications/Delays 28
6.7 Prior and Concomitant Therapy/ Standard of Care 28
6.8 Randomization and Blinding of Study Drug 29
6.9 Maintenance of Study Agent 29
7 Study Procedures 30
7.1 Labs 30
7.2 Established Standard of Care 31
7.3 Treatment Assignment Procedures 31
7.3.1 Randomization Procedures 31
7.3.2 Masking Procedures 31
7.4 Study Visits 31
7.4.1 Screening/Baseline (Day 1): 31
7.4.2 Follow-up 32
7.4.3 Unscheduled 33
7.4.4 Visit 3 34
8 Study Analysis 34
8.1 Sample Size Determination 34
8.2 Statistical Methods 35
8.3 Planned Interim Analysis: 38
9 Data Collection, Handling and Record Keeping 38
9.1 Data Confidentiality 38
9.1.1 Confidentiality of Subject Records 38
9.2 Data Capture 39
9.2.1 Source Documents 39
9.2.2 Case Report Forms 39
9.2.3 Data Collection Tools 39
9.3 Data Management 39
9.4 Data and Safety Monitoring 40
9.5 Records Retention 40
9.6 Specimen Banking 40
10 Assessment of Safety 40

10.1 Specifications of Safety Parameters 40
10.1.1 Definition of Adverse Events (AEs) 40
10.1.2 Definition of Serious Adverse Events (SAE) 41
10.1.3 Definition of Unanticipated Problems (UP) 42
10.2 Classification of an Adverse Event 42
10.2.1 Severity of Event 42
10.2.2 Relationship to Study Agent 42
10.2.3 Expectedness 43
10.3 Time period and frequency for event assessment and follow-up 43
10.4 Reporting procedures 44
10.4.1 Adverse Event Reporting 44
10.5 Study Halting Rules 45
10.6 Safety Oversight 46
10.7 Unblinding Procedure 46
11 Study Monitoring, Auditing, and Inspecting 46
11.1 Medical Monitoring 47
11.2 Protocol Deviations 47
11.3 Auditing and Inspecting 47
11.4 Subject Compliance Monitoring 47
12 Ethical Considerations 48
13 Study Finances 48
13.1 Funding Source 48
13.2 Conflict of Interest 48
14 Publication Plan 48
15 References 48

1. Key Roles

Following is a list of all personnel in key roles:

Principal Investigators:

Nasia Safdar MD, PhD (Lead PI)
University of Wisconsin, Department of Infectious Diseases
UW Medicine Foundation Centennial Building
1685 Highland Ave, Madison, WI, 53705
Email: ns2@medicine.wisc.edu

Mary Beth Graham, MD, PhD (Site PI)
Department of Medicine – Division of Infectious Diseases
Froedtert & Medical College of Wisconsin
9200 W.Wisconsin Ave, Milwaukee WI 53226
Email: mbgraham@mcw.edu

Mayur Ramesh, MD (Site PI)
Henry Ford Hospital
2799 W. Grand Blvd., Detroit. MI 48202
Email: MRAMESH1@hfhs.org

Sahil Khanna, M.B.B.S.
Mayo Clinic
200 1st Street SW
Rochester, MN 55905
Email: Khanna.Sahil@mayo.edu

Scientific Leads: Nasia Safdar MD, PhD, Monika Fischer MD, MSc, L. Silvia Munoz-Price, M.D., Ph.D

Data Coordinating Center: Roger Brown PhD and the UW-Madison Data Coordinating Center

AHRQ Point of Contact: Darryl Gray, MD, Division of Healthcare-Associated Infections

Data Monitoring Committee: Contact: Amy Siedschlag, MS
Data Monitoring Committee Protocol Manager
University of Wisconsin-Madison, UW Institute for Clinical & Translational Research
750 Highland Ave. 2112 Health Sciences Learning Center, Madison, WI 53705
Email: asiedschlag@wisc.edu

Study Monitoring Service: Contact: Kristen Mahaffey
FDA Regulated Research Oversight Program
University of Wisconsin-Madison, UW Institute for Clinical & Translational Research
750 Highland Ave. 2112 Health Sciences Learning Center, Madison, WI 53705
Email: klmahaffey@wisc.edu

2. Background and Introduction

This document is a protocol for a human research study; to be conducted in accordance with Good Clinical Practices (GCP) according to United States Code of Federal Regulations (CFR) applicable to clinical studies; International Conference on Harmonization guidelines; applicable government regulations and Institutional research policies and procedures.

2.1 Background and Scientific Rationale

Introduction *Clostridium difficile (C. difficile)* is a pathogen of major public health importance and the most common cause of healthcare-associated diarrhea, with high rates of recurrent disease, especially in those re-exposed to antimicrobial therapy.[1,2] Data from the Centers for Disease Control and Prevention estimate that in 2011 there were 453,000 incident episodes of *C. difficile* infections (CDI) resulting in 29,300 deaths.[3] The annual cost from CDI in the US is estimated at 1.3 billion dollars.[4]

Over one third of patients who develop CDI will develop one or more recurrences; the risk of subsequent recurrence increases with each episode.[5,6] The main risk factor for initial and subsequent episodes of CDI is antibiotic use. Following completion of therapy for CDI and resolution of an initial episode, recurrent CDI may be triggered when antibiotics are given for other infectious conditions. Preventing CDI in this setting (secondary prevention) is essential to reduce the myriad physical, social, and psychological consequences that accompany recurrent CDI and have been well described by our research group and others. [7,8]

Prevention of recurrent CDI is challenging. Recent interventions such as fecal microbiota transplant or monoclonal antibodies to *C difficile* toxin A are either reserved for patients with multiple recurrent episodes when other treatments have failed, have modes effect size or are prohibitively expensive.[9-11] Recently, the use of oral vancomycin for prophylaxis against recurrent CDI has received attention based on the results of two retrospective cohort studies that showed possible favorable outcomes.[12,13] One study included patients with CDI within prior 6 months who were receiving non-*C. difficile* targeted antibiotics. In the 551 episodes, multivariate analysis showed receipt of oral vancomycin for greater than 50% for the duration of antibiotic treatment was an independent protective factor against recurrent CDI in patients whose initial CDI episode was a recurrence (RR, 0.47; 95% CI, 0.32-0.69; P<0.0001) but not in those with one previous episode of CDI (RR, 0.91; 95% CI, 0.57-1.45; P=0.68). In a second cohort study, 4% of those who received oral vancomycin prophylaxis had recurrent CDI compared to 26 % of those who did not receive vancomycin prophylaxis (OR, 0.12; P<0.001).[13] Although promising, the observational study designs preclude assessment of efficacy because of residual confounding, heterogeneity in data availability, inclusion criteria, study populations and uncertainty regarding the magnitude of the effect. Thus, whether or not oral vancomycin prophylaxis is efficacious, remains an unanswered question. This critical gap in the field needs to be filled because 1) there are few available options for preventing recurrent CDI and 2) oral vancomycin is not without risk, particularly the risk of promoting vancomycin-resistant enterococcus (VRE) gut proliferation. [14-17]

We propose a placebo-controlled double blind randomized trial to evaluate the efficacy of prophylaxis with oral vancomycin for preventing recurrent CDI in patients who have experienced at least one CDI episode in the last 90 days and are receiving antibiotics for a non-CDI indication. Oral vancomycin or placebo will be given for the duration of antibiotic therapy plus 5 days. We will also evaluate the impact of this treatment on the gut microbiota composition and function and VRE gut colonization. We hypothesize that oral vancomycin will reduce the risk of recurrent CDI compared with placebo.

Rationale

Overview: Nearly one third of patient with prior CDI episodes will develop recurrence, especially with concomitant antimicrobial therapy.[18,19] *C. difficile* is the most common cause of hospital-acquired infections [20-22] with half a million Americans afflicted and annual estimates of roughly 29,000 deaths and over 1 billion dollars in healthcare costs.[23] Patients with recurrent CDI report a poor quality of life, prolonged symptoms and marked deterioration in functional ability. [8,24-26] As CDI disproportionately affects older adults (an AHRQ priority population), the adverse consequences of CDI in this patient group are even more pronounced and prolonged.[27-31] Therefore, prevention of CDI, in particular in patients at risk for recurrence is essential.

Scientific premise for Aim 1: To evaluate the efficacy of 125 mg once daily prophylactic oral vancomycin versus placebo to prevent recurrent CDI in patients with at least one CDI episode within the past 180 days and receiving antibiotics for treatment of a non-CDI infection.

Vancomycin is a glycopeptide antibiotic that is well tolerated and not absorbed from the gastrointestinal tract into the bloodstream. It inhibits the cell wall biosynthesis of gram positive bacteria including vegetative forms of *C. difficile* [32] and it is FDA approved as first line treatment for CDI.[33,34,35] **However, the role of oral low dose vancomycin as prophylaxis has not been evaluated**. Because of the effect of vancomycin on *C. difficile*, this approach is biologically plausible and may lead to reduction in the bioburden of *C. difficile*. This line of inquiry is also potentially promising because of 2 recent, observational studies showing a benefit, although the magnitude of benefit varied. [12,13]In a study by Van Hise et al[13], among 203 patients who met eligibility, 35% (n=71) received either 250mg or 125 mg twice daily dose of oral vancomycin treatment concurrently with non-CDI related antibiotic and up to 7 days after non-CDI antibiotic, while 65% (n=132) did not receive oral vancomycin. Compared to the no-vancomycin group, patients on vancomycin had significant reduction in CDI recurrence, even with the lower vancomycin dose. These findings were corroborated in part by Carignan et al[12] who found significantly greater benefit of oral vancomycin prophylaxis in patients with recurrent CDI episodes compared to those with a first CDI episode. Despite this renewed enthusiasm in oral vancomycin prophylaxis, there are no randomized trials that have been completed on this topic (www.clinicaltrials.gov). A randomized controlled trial on this issue is critical because of the limitations of the observational studies including residual confounding, heterogeneity in study populations, differences in outcome ascertainment and follow-up periods and lack of evaluation of potential undesirable consequences of oral vancomycin prophylaxis. Moreover, the uncertainty regarding the magnitude of effect in the observational studies (which can often result in overestimates of efficacy) can be resolved with a randomized trial. A trial is essential to answer the question: Is the benefit of oral vancomycin prophylaxis sufficient to outweigh a potential risk of gut dysbiosis with its use? With the critical importance of antibiotic stewardship, it is important to examine the trade-off between a possible benefit and possible adverse effects such as gut dysbiosis and proliferation of other resistant bacteria such as VRE that may be a collateral undesirable consequence of using oral vancomycin. We therefore propose conducting a double blinded placebo controlled randomized trial (RCT) to evaluate the efficacy and safety of oral vancomycin in preventing recurrent CDI.

Scientific premise for Aim 2: To determine how oral vancomycin prophylaxis alters the gut microbiota, with respect to diversity and function, and if this modification is correlated with a reduction in recurrent CDI.

Vancomycin is not without risk despite its perceived utility in management of CDI episodes. Vancomycin usage has been shown to significantly alter the gut microbiome by decreasing the abundance of Gram-positive bacteria while increasing the abundance of Gram-negative bacteria. [36] Baseline commensals including Bacteroidetes have been dramatically reduced following vancomycin treatment with increases in pathogenic bacteria including *Enterococcus,* and *Clostridium.*[37] Reductions in the overall diversity of the gut microbiota presents opportunities for pathogens such as *C. difficile* to colonize and establish, leading to CDI. However, repeated application of vancomycin can potentially reduce the incidence of recurrent CDI by removing *C. difficile* and allowing for a more normal gut microbiota to establish and thereby preventing future colonization of *C. difficile*. Under this model, we hypothesize that the gut microbiota established after vancomycin treatment is significantly altered from its pre-vancomycin treatment state, and is not only resistant to *C. difficile* colonization, but remains resistant as a result of continued low-doses of vancomycin treatment, relative to a control group. We predict that the gut microbiota of those patients receiving vancomycin treatment will be different with respect to both diversity and function,

relative to the control, and in particular, will feature a microbiota capable of withstanding alterations due to vancomycin use.

Scientific premise for Aim 3: To determine the incidence of gut colonization by VRE in patients receiving intervention vs placebo.

VRE is designated as a “serious threat” by federal research agencies and a major public health challenge both clinically and economically. [38] There are an estimated 20,000 VRE cases and 1,300 associated deaths annually, which account for approximately 30% of all healthcare-associated infections. [38] In addition to the poor clinical outcomes among patients with VRE, there is a significant economic burden due to hospitalization costs from an increased length of stay and the use of more costly antimicrobial agents.[39,40] VRE gut colonization is a prerequisite for VRE infection[41,42] and is associated with 24.6% mortality rate, a 25% increase in total admission days, and a 22% increase in hospital costs compared to non-colonized controls.[43] Oral vancomycin potentially increases the risk of VRE colonization in some but not all studies and is a potential concern with oral vancomycin prophylaxis that has not been addressed. Our proposed study will examine this critical issue.

Scientific premise for Aim 4: To determine if *C. difficile* positivity on any stool sample is a predictor of CDI recurrence.

The clinical impact of *Clostridium difficile* colonization is not yet fully elucidated but data in adults suggest it is an independent risk factor for development of symptomatic *Clostridium difficile* associated diarrhea in those colonized by a toxigenic strain,[43-46] with risk ranging from 14.1-17.9% vs 0.9-1.4% in those not colonized at baseline. In one study of geriatric inpatients in Germany 16.4% of patients were colonized at baseline and 87.5% of cases that developed symptomatic CDI over the study period were known carriers. We will further evaluate the rate of colonization in patients with recent history of treated CDI at baseline and throughout antibiotic treatment for non-CDI indication to elucidate if colonization predicts recurrence, and if that risk of recurrence is decreased by oral vancomycin prophylaxis.

2.2 Hypothesis

We postulate that oral vancomycin will reduce the risk of recurrent CDI compared with placebo and we therefore make the following hypothesis with corresponding specific aims:

<u>Hypothesis 1</u>: a) The proportion of recurrent CDI’s at 8 weeks following completion of oral vancomycin therapy will be significantly lower than placebo. b) Time to recurrence of CDI will be significantly longer in the oral vancomycin group than the placebo group.
<u>Specific aim 1</u>: To evaluate the efficacy of once daily prophylactic oral vancomycin 125 milligrams as secondary prevention versus placebo to prevent recurrent *Clostridium difficile* infection (CDI) in patients receiving antibiotics for treatment of non-CDI indications with history of a CDI episode within the past 180 days

<u>Hypothesis 2</u>: Alteration of diversity and function of gut microbiota will be greater with oral vancomycin administration.
<u>Specific aim 2</u>: Determine how oral vancomycin prophylaxis alters the gut microbiota, with respect to diversity and function, and if this modification is correlated to a reduction in recurrent CDI.

<u>Hypothesis 3</u>: The proportion of VRE incidence at 8 weeks following completion of oral vancomycin therapy will be significantly lower than placebo
<u>Specific aim 3</u>: To determine gut colonization by vancomycin-resistant enterococcus (VRE) in patients receiving intervention vs placebo.

<u>Hypothesis 4:</u> The proportion of patients with *C. difficile* asymptomatic colonization will be at higher risk of CDI recurrence, and oral vancomycin prophylaxis will reduce that risk versus placebo.
<u>Specific Aim 4:</u> To determine if *C. difficile* positivity on any stool sample is a predictor of CDI recurrence.

2.3 Study Agent

Oral vancomycin is an FDA approved tricyclic glycopeptide antibiotic for acute enterocolitis and *Clostridium difficile*-Associated Diarrhea in adults. Oral vancomycin has poor gastrointestinal absorption but effectively inhibits bacterial cell-wall biosynthesis, cell membrane permeability and RNA synthesis. It is stable at room temperature and can be stored at 15-30°C (59-86°F). Oral vancomycin is FDA approved as first line treatment for CDI and is recommended for severe and recurrent CDI. However, the role of oral low dose vancomycin as prophylaxis has not been evaluated. This approach is biologically plausible and can lead to significant gut decolonization of *C. difficile* and reduced prevalence of recurrent CDI.

2.4 Summary of Clinical Data

Two recent retrospective cohort studies reviewed the prophylactic effect of oral vancomycin on preventing recurrent CDI.[12,13] One study evaluated records on adult in-patients on systemic antimicrobial therapy with prior history of loose stool or diarrhea and PCR positive stool test for *Clostridium difficile*. Recurrent CDI was defined as symptomatic loose stool and PCR stool positive *C.difficile* test within 4 weeks of completing the systemic antimicrobial therapy. Of N= 203, 3 (4.2%) patients in the oral vancomycin group compared to 35 (26.6%) in the control group (O.R. 0.12, 95% C.I. 0.04 to 0.4, P<0.0001).[13] Of the 3 CDI cases in the vancomycin group, 2 patients were in the 250 mg and 1 patient was in the 125 mg twice-daily subgroups. Oral vancomycin after systemic therapy averaged 1 day only.

Another study reviewed records on patients with prior history who had received non-CDI related systemic antibiotics within 90 of prior CDI diagnoses. They found higher incidence of recurrence in following first CDI (AHR, 3.59; 95% CI, 2.52–5.13; *P* <0.0001) or a second recurrence (AHR, 4.88; 95% CI, 3.38–7.06; *P* <0.0001). Oral vancomycin prophylaxis (125mg QID) was more effective if CDI was a recurrent episode (AHR, 0.47; 95% CI, 0.32–0.69; *P* <0.0001) compared to a first CDI episode (AHR, 0.91; 95% CI, 0.57–1.45; *P* =0.68). =0.68).[12]

2.5 Dose Rationale

Oral vancomycin with FDA approval for 250 mg or 125 mg dispersion has been evaluated in previous studies. Such studies has indicated prophylactic effects using 125 mg at BID or QID intervals.[12,13] However, 125 mg twice daily regimen indicated better prophylactic effect compared to the 250 mg regimen.[12]

2.6 Potential Risk and Benefits to Subjects

2.6.1 Known Potential Risks

Known side effects from vancomycin range from severe to mild including hearing loss, constipation, nausea, and abdominal pain. However, these adverse effects are more likely to occur with intravenous vancomycin administration. Our study will use an oral administration which is associated with no absorption from the gastrointestinal tract and therefore potentially eliminates the known adverse effects.

Additionally, there is risk of developing antibiotic resistance including vancomycin resistant enterococcus (VRE). We anticipate a lower vancomycin dose of 125 mg once daily will be effective and reduce the risk of developing VRE. However, regular visits and communication between study participants and personnel; and specimen collection and analysis will increase surveillance.

NOTE: in addition to the above, a package insert from FDA website is used as the primary source of risk information.

2.6.2 Protection Against Risks

Oral vancomycin administration has documented poor gastrointestinal tract absorption, which significantly reduces most risks to study participants. To further reduce any risk associated with the study, recruitment and informed consent process will be carried out according to accepted standards and ethical principles; study personnel will be appropriately trained to know what to look for during phone interactions and in-person visits. Any evidence of risk or significant adverse event will lead to withdrawal of the participants and referral for appropriate treatment and follow up. The participant will receive treatment as part of usual care.

2.6.3 Potential Benefits to the Subjects

The risks associated with this study are small. Previous retrospective studies have shown protective effects of oral vancomycin in preventing recurrent CDI. Study participants are more likely to benefit from close health surveillance at follow up consultations in comparison to non-participants. Study findings will help address this critical gap focused on the efficacy of oral vancomycin prophylaxis in preventing recurrent CDI. This study will also provide insight into the effect of oral vancomycin on the fecal microbiota and VRE. If oral vancomycin secondary prophylaxis is found to be efficacious it will shift the current paradigm in the approach to prevention of recurrent CDI.

3 Study Objectives and Purpose

- Primary Objective:
    - To evaluate the efficacy of prophylaxis with oral vancomycin for preventing recurrent CDI.
- Secondary Objectives: -
    - To determine how oral vancomycin prophylaxis alters the gut microbiota, with respect to diversity and function, and if this modification is correlated to a reduction in recurrent CDI.
    - To determine gut colonization by vancomycin-resistant enterococcus (VRE) in patients receiving intervention vs placebo.
    - To determine if *C. difficile* positivity on any stool sample is a predictor for CDI recurrence.

This study will determine the efficacy and safety of prophylaxis with oral vancomycin in patients with history of CDI within the past 180 days who now require non-CDI antibiotic therapy in preventing recurrent CDI. Furthermore, study findings will also provide insight into the effect of oral vancomycin on the fecal microbiota and VRE colonization. With the critical importance of antibiotic stewardship, it is important to examine the trade-off between a possible benefit and possible adverse effects such as gut dysbiosis. If oral vancomycin secondary prophylaxis is found to be efficacious it will shift the current paradigm in the approach to prevention of recurrent CDI.

4 Study Design and Endpoints

4.1 General Design

Schematic of Study Design

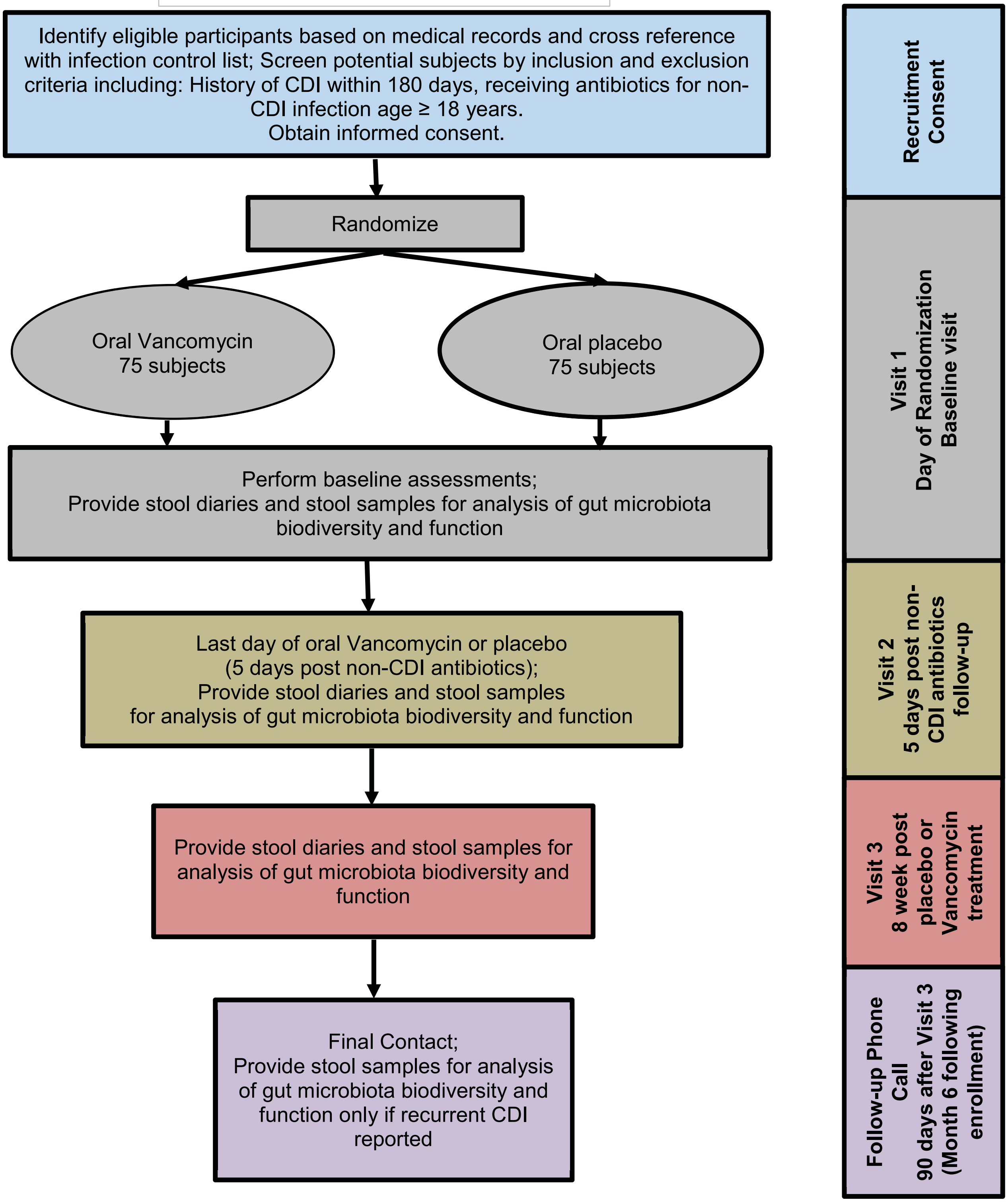

Table 1: Study Visits

A schematic representation of the study visits							
	**Visit 1**<br>Screening, Enrollment and vancomycin or placebo	**Visit 2**<br>+7 days<br>Last dose of vancomycin or placebo	**Visit 3**<br>±14 days<br>8 weeks following last dose of oral vancomycin or placebo	**Weekly phone calls**<br>Until Visit 3	**Early Termination**<br>As needed	**Unscheduled**<br>As needed between enrollment and projected date of Visit 3	**Follow-up Phone Call**<br>±14 days<br>180 days after first dose
Informed consent	X						
Pregnancy test[1]	X						
Physical exam[5]	X	X	X		X		
Vital signs, weight	X[2]	X[2]	X[2]		X[2]		
Height	X						
Demographics	X						
Diet history questionnaire	X						
Significant changes in diet		X	X		X		
Medical history	X	X	X	X	X		
Concomitant medications	X	X	X	X	X	X	X[3]
Adverse events	X	X	X	X	X	X	
CDI recurrence		X	X	X	X	X	X
Stool collection	X	X	X		X	X	X[4]
Patient diaries	X	X	X	X	X		

[1]Women of child-bearing potential only. May be run as urine or blood test. Test performed as part of standard of care within seven days of enrollment is acceptable.
[2] Vital signs, and weight will be completed only if visit is conducted in person.
[3] Only information about CDI-related medications will be collected.
[4] Stool will only be collected in conjunction with follow-up phone call if patient reports a recent recurrence of CDI.
[5] Physical exams will be performed if clinically indicated by patient symptoms per study investigator. Systems examined will depend on patient symptoms.

Screening/Baseline: Hospitalized patients or those in the ambulatory setting with a recent history of at least one episode of CDI (within the past 180 days) and currently on oral or IV antibiotic treatment for a non-CDI related indication with anticipated duration of no more than 2 weeks will be approached for participation. Participants will be within 72 hours of commencing non-CDI antibiotic therapy. Study goals and procedures will

be discussed and all concerns addressed. Only after signed informed consent has been obtained will the participant be enrolled and randomized into the study by the research team. The following data will be collected by chart review and subject questionnaire:

Demographics including date of birth, sex, race, and ethnicity; medical history and allergies, comorbidities, current medication list, antibiotic status and use in last 180 days, details of current non-CDI infection, social history, dietary history using a diet questionnaire[44], physical exam if clinically indicated, vital signs (blood pressure, pulse, temperature), height, weight, body mass index, current bowel habits using a modified Bristol stool scale[45, 46]. The physical and/or vital signs may be extracted from the patient's medical record if all data consistent with study form requirements is available at a time point within two weeks prior to enrollment. Patients will begin dosing with oral vancomycin or placebo after all pre-dose procedures have been completed. The diet questionnaire does not need to be completed prior to dose

All outpatients will be strongly encouraged to attend Visit 1 in person. If they are unable to do so and have capacity to consent, no in-person visit is required. Study capsules will be shipped to patients per local site policies. Study capsules may be mailed prior to collection of a stool sample.

Visit 2: Oral vancomycin or placebo will continue for additional 5 days after completing non-CDI antibiotic. Upon completing oral vancomycin or placebo, the study team will perform a clinical assessment of the participant and administer a study questionnaire which will specifically inquire about solicited and unsolicited symptoms of AEs. Access to the medical records may be utilized to capture salient changes in healthcare status. Participants will provide a stool sample at second visit (+7 days). In person follow-up is preferred for this visit but it may be conducted via phone call if participant is unable to return to clinic. If visit is completed via phone call, participant will not have vital signs body weight or physical examination performed. If the subjects chooses not to return in person, they will be asked to return any remaining study drug and stool sample by mail.

Visit 3 : 8 weeks (± 14 days) after completing vancomycin or placebo therapy, the study team will perform a clinical and safety assessment of the participant specifically evaluating adverse events related to the intervention. Data collection at visit will include: clinical evaluation and medical history; concomitant medication including antibiotics; significant changes in diet; any changes in stool consistency and frequency; general health status; any adverse events. Participants will also provide a stool sample during this visit. This visit may be conducted via phone call if participant is unable to return to clinic. If visit is completed via phone call, participant will not have vital signs body weight or physical examination performed. However, we will ask that they collect and send a stool sample by mail. Participants will be asked to call the study team if they have a recurrent CDI prior to the Month 6 phone call.

Early termination visit: Participants who have a recurrent CDI prior to Visit 3 or end their participation for any reason prior to Visit 3 will be asked to return to the study site for medical and safety assessment and stool sample collection. If the subjects chooses not to return in person, they will be asked to return any remaining study drug and a stool sample by mail.

Unscheduled: At the time of CDI recurrence (for participants who experience an infection), study staff will collect a stool sample, and complete an infection CRF for each CDI. This CRF will be used to track duration of the episode, type, dosage and duration of antibiotics. Data points for infectious episode will include: date, preliminary diagnosis, antibiotic/s [Name, Formulation (IV, suspension, capsules), Dosage, Prescribed duration, Final (actual) duration of antibiotics, Change in antibiotics regimen, Concomitant medication (Name, dose, route of administration, duration)]. For subjects who experience recurrent CDI, stool samples will be collected as soon as possible. If the CDI recurrence occurs prior to Visit 3, an early termination visit should also be completed. The subject's participation in the study will end once the unscheduled and early term procedures (if applicable) are completed. The participant's chart will be followed until resolution of CDI to track infectious episode data points but no further contact with the subject will be made.

Weekly phone calls: Weekly phone calls will be undertaken through Visit 3 for ambulatory patients to encourage medication adherence, review stool consistency/frequency, and inquire about adverse effects. For hospitalized patients, research staff will undertake weekly visits until discharge.

Passive chart review (between Visit 3 and Month 6 phone call): Study staff will review patient medical record throughout each week to assess for recurrence of CDI. No AEs or SAEs other than CDI will be collected past Visit 3.

<u>Month 6 phone call:</u> Patients will be called to inquire about recurrence of CDI. Only CDI and related antibiotic use will be collected during this time period; no other data points will be collected.

4.1.1 Primary Study Endpoint:

- Episodes of CDI recurrence during vancomycin therapy and in 8 week period following completion of study interventions in patients receiving vancomycin vs placebo.

4.1.2 Secondary Study Endpoints:

- Changes in biodiversity and function of the gut microbiota between the treatment and comparator group at Visit 3.
- Changes in gut colonization by vancomycin-resistant enterococcus (VRE) in patients receiving intervention vs placebo group at Visit 3.
- To determine if *C. difficile* positivity on any stool sample is a predictor for CDI recurrence.

4.1.3 Primary Safety Endpoint:

Using structured CRFs at scheduled visits as well as weekly phone calls, we will inquire about potential adverse effects of oral vancomycin use. These may include nausea, abdominal pain and bloating. The frequency of adverse events will be compared between the active treatment group and placebo group. In addition, if VRE infection occurs in a study participant, additional stool samples may be collected as well as the isolate causing the infection to evaluate if colonization by VRE was present at or before the infection. In addition, we will follow FDA and AHRQ guidance on safety assessment of the intervention.

Participants will be assessed for:

a) Clinical and safety assessment at baseline (first visit) day 1 before the first dose of oral vancomycin or placebo is ingested. This will use a structured case report form (CRF) at an in-hospital assessment or telephone assessment if the patient is discharged.

b) Clinical and safety assessment after last dose of vancomycin or placebo (+7 days). This will use a structured CRF at an in-hospital assessment, clinic or a telephone assessment if the patient is discharged.

c) Clinical and safety assessment at week 8 (±14 days) after last dose of vancomycin or placebo. This will use a structured CRF at an in-hospital assessment, clinic or a telephone assessment if the patient is discharged.

d) Information regarding the co-administration of any oral or parenteral antimicrobial agents or probiotics, any acid blockers (H2 blockers and PPIs) or any antiperistaltics will be recorded through Visit 3.

Stool samples will be collected at:

a) Enrollment (predose)

b) Visit 2: Occurs after last dose of vancomycin or placebo. (+7 days)

c) Visit 3: Occurs 8 weeks after last dose of vancomycin or placebo. (±14 days)

d) If a subject develops VRE infection, a sample will be collected.

e) Stool will be collected if a subject exhibits symptoms defined as recurrence of CDI.

f) At the time of Early Termination (if applicable).

5 Study Subjects – Enrollment and Withdrawal

Definitions:

Recurrence of CDI: Clinical recurrence is defined by the reappearance of three or more diarrheal stools (grades 5 to 7 on the Bristol Stool Scale [45,46]) per 24-hour period while on study treatment or within the study follow-up period after completion of oral vancomycin or placebo; *C. difficile* toxin A or B, or both, in stool; and a need for retreatment for *C. difficile* infection. If PCR testing is done, toxin testing needs to be positive regardless of PCR results.

VRE colonization: A positive stool test for VRE using selective microbiologic media.

5.1 Subject Population

- The eligibility criteria will provide a definition of participant characteristics required for study entry/enrollment.
- This multi-center, randomized, double blind, placebo-controlled, parallel group trial will compare the efficacy and safety of oral vancomycin to prevent recurrent CDI. It will be performed in collaboration with Medical College of Wisconsin, Henry Ford Hospital, and University of Wisconsin (UW).
- Prior laboratory confirmation of CDI will be used for screening purposes. Stool assessments will be used to identify VRE colonization prior to enrollment and during follow-up.
- Participants will be 18 years of age or older with at least one episode of CDI in the last 180 days and receiving systemic antibiotics for a non-CDI indication. Participants may be hospitalized or in the ambulatory setting. We have chosen the study sites carefully to allow sufficient enrollment of persons of diverse racial/ethnic backgrounds.
- Participants must meet inclusion criteria and have no exclusion criteria prior to randomization.
- Subjects will then be randomized in a 1:1 allocation ratio to either the intervention or control group.
- There will be no enrollment restrictions based upon race or ethnic origin. All participants will have to meet the study criteria to be included in the study. Within the limitations imposed by the population of our study site(s), we would strive to include sufficient enrollment of persons of diverse racial/ethnic backgrounds to ensure that the benefits and burdens of research participation are distributed in an equitable manner.

5.2 Subject Screening for Recruitment

5.2.1 Inclusion Criteria

Inclusion Criteria	
1	Patient and/or legally authorized representative is willing to provide written informed consent
2	Patient and/or legally authorized representative and/or caregiver is willing to comply with all study procedures and be available for the duration of the study
3	Male or female, at least 18 years of age
4	Documented diagnosis of at least one CDI within the last 180 days with treatment completed
5	Currently receiving systemic antibiotics for a non-CDI indication with anticipated duration of no more than 2 weeks[1]
6	Females of childbearing potential must have a negative pregnancy test prior to randomization and agree to use adequate contraception (hormonal or barrier method of birth control; abstinence) prior to randomization, for the duration of study participation, and for 4 weeks following completion of study treatment.<br>A female of child-bearing potential is any woman (regardless of sexual orientation, having undergone a tubal ligation, or remaining celibate by choice) who meets the following criteria:

	• Has not undergone a hysterectomy or bilateral oophorectomy; or<br>• Has not been naturally postmenopausal for at least 12 consecutive months (i.e., has had menses at any time in the preceding 12 consecutive months)
7	Have received no more than 72 hours of non-CDI antibiotics[2]

[1] Concurrent antibiotic use is acceptable if the antibiotics are unlikely to significantly contribute to an active C. diff infection and are not intended to treat C. difficile per investigator discretion. Patients may enroll if antibiotic duration will be more than 14 days if the duration is due to administrative or logistical factors (e.g. scheduling) rather than an infection that requires an extended course of antibiotics. Patients will not be eligible if the non-CDI antibiotic duration exceeds the maximum study dose length (19 days).

[2] Long-term antibiotic therapy (if applicable) is not subject to the 72-hour window referenced in inclusion #7.

5.2.2 Exclusion Criteria

**Exclusion Criteria**
1. History of hypersensitivity or allergy to oral vancomycin
2. Current use of oral vancomycin (Patients placed on oral vancomycin prophylaxis may be enrolled if they have received no more than three doses and are willing to discontinue use)
3. Patients who are on concurrent treatment with metronidazole or tetracycline monotherapy for any indication
4. Diagnosed with bacterial gastrointestinal infection caused by agents other than *C. difficile* that causes diarrhea (e.g. *Salmonella* sp.), toxic megacolon and/or known small bowel ileus
5. Inability (e.g. dysphagia) to or unwilling to swallow capsules
6. Major gastrointestinal surgery (e.g. significant bowel resection) within 3 months before enrollment. This does not include liver/pancreas transplants, appendectomy or cholecystectomy
7. History of total colectomy or bariatric surgery
8. Unable or unwilling to comply with protocol requirements
9. Expected life expectancy < 6 months
10. Received investigational drugs within 30 days prior to randomization
11. Women who are pregnant or breast-feeding
12. Not suitable for study participation due to other reasons at the discretion of the investigator
13. Suspected *C.diff* infection as evidenced by unexplained diarrhea (3 or more loose stools in a 24 hour period) at enrollment. Patients who have diarrhea must have a negative *C.diff* test within 72 hours of enrollment in order to enroll.

5.3 Subject Identification

Subject recruitment will involve site inpatients and ambulatory patients with a recent (180 day) history of CDI. These participants must be within 72 hours of having started systemic antibiotics for a non-CDI related illness. Any systemic antibiotic except those listed in exclusion criteria qualifies for inclusion given the vast number of antibiotics that can be associated with increased *C. difficile*. There will be no difference in subject recruitment per intervention group. The PI and the study research personnel will be able to approach patients within 72 hours of having started antibiotics for screening and recruitment. Participants will be encouraged to ask questions during conversations regarding the study objectives and plans.

5.3.1 Recruitment and Retention Strategies

Adult inpatients that are on infectious control list secondary to a recent (180 day) CDI diagnosis and potentially meet the inclusion criteria will be invited to join the study. If patients have been discharged before recruitment, we will utilize the 'mailed letter option' signed by a study provider with clear study descriptions to reach them and schedule a day/time for a phone screening. If the study team identifies an outpatient through screening that has not previously been contacted and appears to be potentially eligible, a study team member will cold

call them (using specific telephone script) to assess their interest in the study without mailing a letter first due to the 72-hour enrollment window. If they are interested they will be invited to participate in further screening and possible study enrollment. Patients may also be sent a text message with IRB approved language. If a patient requests more information after receiving a text message further texts may be sent by the study team. HIPAA privacy rules must be followed.

Potentially eligible patients will also be recruited from ambulatory settings. Clinic providers will be given information on the study and eligibility criteria in order to assess patient's interest in the study. Interested patients will be contacted by a study team member to further discuss the study and meet for the enrollment visit if indicated.

Inpatients who have a diagnosis of CDI within the past 180 days will be approached by the study team to introduce the study and provide contact information for the study should the patients be prescribed antibiotics not targeting CDI. The mailed letter option may also be utilized for those with known CDI within the past 180 days to alert them to the study with contact information should they be prescribed antibiotics and be interested in joining the study.

One method research teams will use to identify potentially eligible patients from ambulatory settings is through viewing schedules of clinics that have a high percentage of potential subjects. These include Infectious Disease, Transplant and Primary Care clinics and may expand as other focus areas are identified. Patients from these clinic lists will be prescreened for eligibility and approached as previously described. This prescreening process will help ensure only patients who are likely to meet eligibility criteria are approached.

Advertising directed at patients will be used for this study. Examples include, but are not limited to, flyers and digital display ads in hospitals/clinics affiliated with the study sites, and web advertising. All patient-facing recruitment ads/materials will be approved by the IRB prior to use.

If available, individual sites are permitted to use software (e.g. Clinithink) as screening aids provided they meet standards for data confidentiality as outlined in the protocol.

Of the total screened, 150 patients will be enrolled into the study from study sites including UW Hospital. Additional patients will be enrolled as needed until 150 fully evaluable patients (complete study through Visit 3 or are removed due to CDI recurrence) are completed. This study will use a competitive enrollment model. Given the location of the UW hospital in the Midwest, we anticipate a majority Caucasian participant population; nonetheless, efforts will be made to increase participation from minorities, particularly from MCW. Recruitment will be conducted by PI and research staff, and there will be no difference in recruitment methods between the intervention and control groups.

5.4 Vulnerable Populations

TABLE 2: Vulnerable populations

Include	Exclude	Vulnerable Population Type
x		Adults unable to consent
	x	Individuals who are not yet adults (e.g. infants, children, teenagers)
	x	Wards of the State (e.g. foster children)
	x	Pregnant women
	x	Prisoners

5.5 Subject Capacity

Informed consent will be obtained from patients who have capacity to provide informed consent. Legally authorized representatives (LARs) can sign for patients who do not have capacity to consent. The study will be explained to the patient to the extent feasible to ensure patient assent. If a patient regains the ability to consent while on study they will be consented at that time. Patients will be presumed to be able to consent unless there is evidence to the contrary.

UW's Health Sciences IRB policy for "Research with Adult Participants Lacking Capacity to Consent" will be followed. See https://kb.wisc.edu/hsirbs/29545 for further details.

5.5.1 Subject Comprehension

Participants will be allowed adequate time to read study materials, and ask follow up questions. Study teams will encourage participants to summarize their understanding of the study objectives and participant involvement.

5.6 Informed Consent

The PI will be responsible for ensuring that valid consent is obtained and documented for all subjects unless the IRB waives the requirement for documentation of informed consent for all or part of the study.

The consent process will be in person or via telephone. Initial contact in the clinic or inpatient setting will come from clinical team involved in a patient's care or the study site PI. Outpatient telephone recruitment will be preceded by a recruitment letter except if a patient is fully eligible, to meet the time restriction of enrolling within 72 hours of starting a non-CDI antibiotic.

5.6.1 Process of Consent

The written consent document will embody the elements of informed consent as described in the Declaration of Helsinki and will adhere to the ICH Harmonized Tripartite Guideline for Good Clinical Practice. Informed consent should be implemented before any protocol-specified procedures or interventions are carried out. Informed consent will be obtained in accordance with 21 CFR 50.25 and 45 CFR 46. Information should be presented both orally and in written form.

An investigator or designee will describe the protocol to potential participants. The Participant Information and Consent Form may be read to the participants, but, in any event, the investigator shall give the participants ample opportunity to inquire about details of the study and ask any questions before the signing and dating the consent form.

Study staff must inform participants that the trial involves research, and explain the purpose of the trial, those aspects of the trial that are experimental, any expected benefits, all possible risks (including a statement that the particular treatment or procedure may involve risks to the participant or to the embryo or fetus, if the participant is pregnant or may father a child, that are currently unforeseeable), the expected duration of the participant's participation in the trial, the procedures of the research study, including all invasive procedures, and the probability for random assignment to treatment groups. Participants will be informed that they will be notified in a timely manner if information becomes available that may be relevant to their willingness to continue participation in the trial. They must also be informed of alternative procedures that may be available, and the important potential benefits and risks of these available alternative procedures. Participants must receive an explanation as to whether any compensation and any medical treatments are available if injury occurs, and, if so, what they consist of, or where further information may be obtained. Participants must be informed of the

anticipated financial expenses, if any, to the participant for participating in the trial, as well as any anticipated prorated payments, if any, to the participant for participating in the trial. They must be informed of whom to contact (e.g., the PI or study physician/nurse practitioner) for answers to any questions relating to the research project. Information will also include the foreseeable circumstances and/or reasons under which the participant's participation in the trial may be terminated. The participants must be informed that participation is voluntary and that they are free to withdraw from the study for any reason at any time without penalty or loss of benefits to which the participant is otherwise entitled.

Neither the investigators, nor the trial staff, should coerce or unduly influence a participant to participate or continue to participate in the trial. The extent of the confidentiality of the participants' records must be defined, and participants must be informed that applicable data protection legislation will be followed. Participants must be informed that the monitor(s), auditors(s), IRB and regulatory authority(ies) will be granted direct access to the participant's medical records for verification of clinical trial procedures and/or data without violating the confidentiality of the participant, to the extent permitted by the applicable laws and regulations, and that, by signing a written informed consent form, the participant is authorizing such access. Participants must be informed that records identifying the participant will be kept confidential, and, to the extent permitted by the applicable laws and/or regulations, will not be made publicly available and, if the results of the trial are published, the participant's identity will remain confidential.

5.6.2 Consent Form

Consent forms must be in a language fully comprehensible to the prospective participants. Informed consent shall be documented by the use of a written consent form approved by the IRB and signed and dated by the participant and the person who conducted the informed consent discussion. The signature confirms that the consent is based on information that has been provided and all questions have been answered to the prospective participant's satisfaction. Each participant's signed informed consent form must be kept on file by the investigator for possible inspection by Regulatory Authorities and/or the sponsor and Regulatory Compliance persons. The participant should receive a copy of the signed and dated written informed consent form and any other written information provided to the participants, and should receive copies of any signed and dated consent form updates and any amendments to the written information provided to participants.

5.6.3 HIPAA

Information about study subjects will be kept confidential and managed according to the requirements of the Health Insurance Portability and Accountability Act of 1996 (HIPAA).

5.6.4 Revoking Consent

In the event that a subject revokes authorization to collect or use PHI, the investigator, by regulation, retains the ability to use all information collected prior to the revocation of subject authorization. For subjects that have revoked authorization to collect or use PHI, attempts will be made to obtain permission to collect at least vital status (i.e. that the subject is alive) at the end of their scheduled study period.

5.6.5 Costs to the Subject

No charge may be made to subjects as the costs are covered by a grant.

5.6.6 Payment for Participation

Up to $300 will be given to subjects for participation.
This amount will be prorated if a subject drops out earlier according to the following schedule:

a. Completion of enrollment $150
b. Completion of Visit 2 $50
c. Completion of Visit 3 $50
d. Completion and return of all patient diaries $50

5.7 *Early Withdrawal of Subjects*

5.7.1 Premature termination of study

This study may be temporarily suspended or prematurely terminated if there is sufficient reasonable cause. Written notification, documenting the reason for study suspension or termination, will be provided by the suspending or terminating party to investigator, funding agency, and regulatory authorities. If the study is prematurely terminated or suspended, the PI will promptly inform the IRB and will provide the reason(s) for the termination or suspension.

Circumstances that may warrant termination or suspension include, but are not limited to:

- Determination of unexpected, significant, or unacceptable risk to participants
- Demonstration of efficacy that would warrant stopping
- Insufficient compliance to protocol requirements
- Data that are not sufficiently complete and/or evaluable
- Determination of futility

Study may resume once concerns about safety, protocol compliance, data quality are addressed and satisfy the sponsor, IRB and/or FDA.

5.7.2 When and How to Withdraw Subjects

A participant may withdraw from the study at any time for any reason, without any consequence. A participant may be withdrawn from the study by the Investigator for the following reasons:

- Adverse event that may make it no longer in the best interest of the subject to continue participation in the study
- Participant choice (withdrawal of consent)
- Protocol violation/non-compliance
- Pregnancy
- Lost to follow up
- Other (must be noted)

5.7.3 Data Collection and Follow-up for Withdrawn Subjects

The primary reason for withdrawal from the study will be recorded. Participants will be encouraged to complete the Early Termination Visit. The Early Termination Visit procedures are listed in Section 7.4.2. Although participants are free to withdraw at any time, subjects will be encouraged to remain in the study for follow-up safety evaluation. Every attempt should be made to follow all AEs and SAEs ongoing at the time of early withdrawal to resolution or until stabilized. If possible, a final stool sample will be collected at the Early Termination Visit.

If a subject withdraws prior to study Visit 3, an additional subject will be enrolled until 150 subjects complete all dosing/follow up visits. All subjects who are randomized will be included in the intention to treat statistical analysis.

Recurrence of CDI is a reason for withdrawing a participant from the study after randomization, as defined in Section 5.

6 Study Agent

6.1 Description and Formulation

See section 2.3 above

6.2 Packaging

Study drugs will be packaged in 125 mg capsules with labeling and dosage instructions. Appropriate amounts of either vancomycin or placebo will be given to participants after randomization. We anticipate patient will receive 19 days' supply of the study intervention. Subjects who have completed non-CDI dosing prior to enrolling, and those receiving one dose of non-CDI antibiotic will be distributed 5 days of study medication per investigator discretion, dependent on local site pharmacy capabilities.

6.3 Preparation, Administration and Storage of Study Drug

The drug is stored, mixed/prepared or dispensed from each site pharmacy research center (PRC). Placebo will be as indistinguishable from vancomycin as possible. If placebo is not sufficiently similar to oral vancomycin to be indistinguishable then study staff will not perform any activities that involve handling oral vancomycin or placebo pills to ensure treatment blinding. The site PRC will dispense the medications to the participants following randomization and enrollment. All investigational agents must be processed through each site's PRC.

6.4 Route of Administration

Oral vancomycin and placebo will be used.

6.5 Starting Dose and Dose Escalation Schedule

There are no dose adjustments as part of this study protocol (or standard of care). A maximum of 19 days of oral vancomycin/placebo will be dispensed to each subject.

6.6 Dose Adjustments/Modifications/Delays

There is no drug washout/taper period, and current medication will not be withheld during the treatment. If a dose is missed, participants will be advised to record the missed dose in their diary and resume the next day if further dose(s) are scheduled.

Modifications in length of oral vancomycin/placebo will occur if the duration of a participant's non-CDI antibiotic is changed. If a non-CDI antibiotic course is shorter than expected at time of enrollment, patients will take oral vancomycin/ placebo for five days post non-CDI antibiotics. If a non-CDI antibiotic course is lengthened the patient's oral vancomycin/placebo will be extended to have the patient take study pills for five days post non-CDI antibiotics, up to a maximum of 19 study doses.

If a patient completed their non-CDI antibiotics prior to first dose of study capsule (i.e. single dose non-CDI antibiotic), study vancomycin/placebo will be administered for a total duration of five days.

If post-dose vomiting occurs and the capsule is visualized in the emesis, the subject should take another dose.

6.7 Prior and Concomitant Therapy/ Standard of Care

Given that study participants are adults with CDI and patients who are most at risk for recurrent CDI are those with multiple comorbidities, all medications will be accepted in this study except those listed under exclusion criteria, at the discretion of the study staff. This includes, but is not limited to:

1. Anti-hypertensive therapies

2. Heart failure medications
3. Diuretics
4. Laxatives
5. Topical medications
6. Anti-depressants
7. Statins
8. Diabetic medication
9. NSAIDs
10. Acid-blockers
11. Anticoagulants
12. Opioids
13. Iron supplementation
14. Anti-TNF will be permitted. Patients on monoclonal antibodies to B and T cells. Glucocorticoids, antimetabolites (azathioprine, 6-mercaptopurine, methotrexate), calcineurin inhibitors (tacrolimus, cyclosporine) and mycophenolate mofetil may be enrolled only after consultation with the medical monitor.
15. Antibiotics administered via inhalation with limited systemic absorption (i.e. nebulized tobramycin)

6.8 Randomization and Blinding of Study Drug

Participants, who fulfill the criteria for enrollment, will be randomized on a 1:1 block scheme (75 subjects per arm) to receive oral vancomycin or placebo. Participant's randomization will be carried out by Dr. Brown (bioinformatician) using random number generation software to create a randomization schedule.

We proposed to use a permuted block randomization instead of simple stratified randomization. Subsequently, study subjects are divided into a large number of blocks, typically a small block of 2 or 4, and simple randomization is done in each block. This method can ensure the approximate balance at end of recruitment, minimize chance of imbalance due to unexpected shortfall in enrollment and facilitate planning and implementation of the treatment administration process. A drawback of this method is that once the first few assignments in a block are revealed future assignments will become known or predictable. To compensate, a variation of the stratified permuted-block randomization that randomly mixes blocks of different sizes has been widely used to overcome this problem. Our stratified variable permuted-block randomization will allocate treatments labelled "Placebo" and "Active" equally in two randomly presented block sizes, 2 and 4, to 200 subjects in each of 4 strata. We will use Stata Version 15 'ralloc' routine to obtain the randomization. These stratified random files may be easily incorporated into REDCap.

This is a double-blinded placebo-controlled trial where both the participant and study staffs will be blinded. The oral vancomycin or placebo will be dispensed by participating site Pharmaceutical Research Center (PRC), thereby maintaining blinding of study clinician. Packaging of vancomycin and placebo will be identical to retain blind.

Under normal circumstances, the blind should not be broken. The blind should be broken only if specific emergency treatment would be dictated by providing the treatment the participant was receiving. In such cases, the Investigator must contact the Medical Monitor to request that the blind be broken. For participants who require unblinding, this information will be captured in the case report form (CRF).

The randomization schedule will be maintained by study team. Study participants will remain blinded and not be provided any information until all participants have completed the trial.

6.9 Maintenance of Study Agent

Table 3: Study Agent information at a glance

Name	***Source***	***Storage***	***Dispensing***	***Disposal***

Vancomycin	***Site PRC***	***Site PRC***	***Site PRC***	***Site PRC***
Placebo	***Site PRC***	***Site PRC***	***Site PRC***	***Site PRC***

Each site's Pharmaceutical Research Center (PRC) will manage the distribution of vancomycin and placebo. If shipped from a manufacturer, an inventory will be performed and a drug receipt log filled out and signed by the PRC staff accepting the shipment. The designated staff will count and verify that the shipment contains all the items noted in the shipment inventory. Any damaged or unusable study drug in a given shipment (active drug or comparator) will be documented in the study files. The investigator will notify the study sponsor of any damaged or unusable study treatments that were supplied to the investigator's site. PRC will oversee storage and dispensing of the study interventions. Study staff will notify PRC of any order signed by the study physician with patient's information.

At the completion of the study, there will be a final reconciliation of drug shipped, drug consumed, and drug remaining. This reconciliation will be logged on the drug reconciliation form, signed and dated by PRC and study staff. Any discrepancies noted will be investigated, resolved, and documented prior to destruction of unused study drug. Drug destroyed on site will be documented in the study files.

The site principal investigator (or designee) will maintain an accurate record of the receipt of the investigational materials as shipped by the sponsor (or designee), including the date received. One copy of this receipt will be returned to the sponsor when the contents of the investigational materials shipment have been verified. This clinical trial material accountability record will be available for inspection at any time.

7 Study Procedures

7.1 Labs

Stool collection: This study will include 3 scheduled stool samples and/or perirectal swabs if stool sample(s) cannot be produced. A baseline stool sample (or perirectal swabs) will be collected at enrollment and at Visit 2 (+7 days) and Visit 3 (±14 days). Stool specimens will be self-collected by participants using a provided commercial "toilet hat" stool collection kit, including cold packs. Our current studies use this protocol and we have had over 95% adherence to subjects' self-collection and shipping of specimens in the correct containers and conditions. We will use the protocols recommended by the Human Microbiome Project.[47] Processing of stool specimens will occur separately for microbiota and conventional microbiology analysis. Time between specimen collection and extraction has been examined as a possible confounder by the Human Microbiome Project, and no bias was observed.[52] Microbiota analysis will be performed at the UW. Specimens will be stored at -80 degrees Celsius at the UW lab for microbiota analysis and will be processed immediately for microbiologic analysis. The findings will be communicated to patients, but may not be in the electronic health record. Stool may also be collected if concern for VRE colonization or recurrence of *Clostridium difficile* arise, or early withdrawal from the study with the subject's consent.

When recurrence is suspected each site will evaluate, test and treat per protocol and stool will also be sent to UW for culture for *C. difficile, C. difficile* toxin, *C. difficile* PCR and UW will hold stool to determine if cytotoxin assay is needed on a case by case basis.

Microbiologic Analysis. To determine the presence of *C. difficile* and VRE in qualitative stool cultures, broth enrichment followed by culture onto selective agar will be used. Toxin detection will be done using TechLab *C. difficile* TOX A/B II. Suspected VRE and *C. difficile* isolates will be speciated by Sanger Sequencing for the 16S rRNA gene. Toxin detection cannot be performed on perirectal swabs.

Patient diaries: The patient diary will include both an oral vancomycin/placebo dosing diary and a stool diary. The modified Bristol stool consistency scale will be used for assessment of diarrhea. This scale has been extensively used and validated, and we have experience with it in a previous trial. [48] The subjects will fill out a

stool diary from baseline until Visit 3. Subjects will be asked to record the consistency, date and time of each stool they pass. The research team will train the subject on how to complete it. Numbers 5-7 on the scale are considered consistent with diarrhea.

7.2 Established Standard of Care

There are currently no established standards of care.

7.3 *Treatment Assignment Procedures*

7.3.1 Randomization Procedures

Participants who fulfill the criteria for randomization will be randomized on a 1:1 allocation ratio to receive oral vancomycin or placebo. Participant's randomization will be carried out by our biostatistician based at the UW coordinating center using random number generation software to create a randomization schedule.

7.3.2 Masking Procedures

This is a double-blinded placebo controlled trial where both the participant and study staff will be blinded. The oral vancomycin or placebo will be dispensed by site pharmacy thereby maintaining blinding of study clinician. Packaging will be identical and consist of the same saline and glycerol buffer used in vancomycin processing.

Under normal circumstances, the blind should not be broken. The blind should be broken only if specific emergency treatment would be dictated by providing the treatment the participant was receiving. In such cases, the site must contact the Medical Monitor to request that the blind be broken. For participants who require unblinding, this information will be captured in the case report form (CRF).

The randomization schedule will be maintained by Madison, WI center. Study participants will remain blinded and not be provided any information until all participants have completed the trial.

7.4 Study Visits

(Schematic representation in section 4.1 above)

Study visits will be conducted in outpatient clinic rooms or inpatient rooms (if applicable). At the UW, we will also be utilizing the Clinical Research Unit (CRU) for conducting consenting and study visits.

7.4.1 Screening/Baseline (Day 1):

Patients with a recent history of CDI (within the past 180 days) and currently on antibiotic treatment for a non-CDI related ailment will be approached by a member of his/her clinical team to request that the research team may discuss the study. Study goals and procedures will be discussed and all concerns addressed. Only after signed informed consent has been obtained will the participant be enrolled and randomized into the study by the research team.

If a patient with a recent history of CDI (within the past 180 days) is prescribed antibiotic treatment for a non-CDI related ailment and agrees to participate in the study but has not yet started taking the antibiotic treatment, the study team may dispense a stool sample collection kit to the prospective participant with a waiver of consent documentation. The potential participant will be instructed to collect a stool sample within three days of their scheduled initial dose of antibiotic treatment.

- Data points at enrollment study visit:
  - General parameters:
    - Date of birth

        - Sex
        - Race
        - Ethnicity
    - Past medical history and allergies
        - Comorbidities
    - Current medication list
    - Antibiotic status and use in last 180 days
    - Details of current non-CDI infection
    - Dietary history, using a validated questionnaire[44] (may be collected after dosing)
    - Social history
        - Alcohol, smoking status
    - (Vital signs: temperature, height, weight (height/weight measured or abstracted from medical records if taken within last year), heart rate, blood pressure. May be transcribed from medical record if performed within two weeks of enrollment visit.
    - Physical exam: relevant review of systems if clinically indicated
    - Current state of bowel movements- using a validated visual stool instrument

- Patient is eligible for randomization after all required enrollment procedures (except stool collection and diet questionnaire) have been completed.
- Sample collection (collected using a stool collection kit/hat or peri-rectal swab) must be collected prior to patient taking first study dose of vancomycin/placebo. Results of predose stool sample are not needed prior to dose unless required for determination of exclusion due to diarrhea.

After baseline clinical asssessment and stool sample have been collected, the participant is eligible to begin dosing. Participants will be provided with information regarding monitoring for minor and severe adverse event related to the intervention administration. Participants will be encouraged to report any concerning symptoms to study staff.

7.4.2 Follow-up

The study team will perform a clinical assessment and administer a study questionnaire which will specifically inquire about solicited and unsolicited symptoms of AEs. Access to the medical records may be utilized to capture salient changes in healthcare status. Participants will submit a stool sample within each follow-up window.

During the 2nd and 3rd visits participants will undergo a similarly structured study visit which includes a clinical assessment and AE focused questionnaire.

Visit 2 Completion of vancomycin or placebo (+7 days)

The study physician/nurse/coordinator will perform a clinical assessment and administer a study questionnaire which will specifically inquire about solicited and unsolicited symptoms of AEs. Access to the medical records may be utilized to capture salient changes in healthcare status. Participants will have a stool sample within each follow-up window.

- Data collection at visit:

    - Interim medical history with focus on infectious diseases

  - Concomitant medication
  - Significant changes in diet
  - Changes in stool consistency (Bristol Stool Scale) and frequency
  - Vital signs (if visit performed in-person): Temperature, heart rate, blood pressure, weight
  - Adverse events (NIH criteria)
  - Stool collection or peri-rectal swabs

Early termination visit

In the case of an early termination, study staff will complete an ‘Early Termination CRF’, if possible, and the following will be assessed:

- Clinical and safety assessment by study team, specifically evaluating adverse events related to the intervention.
- Data collection at visit:
  - Interim medical history with focus on infectious diseases
  - Concomitant medication
  - Significant changes in diet
  - Changes in stool consistency (Bristol Stool Scale) and frequency
  - Vital signs (if visit performed in-person): Temperature, heart rate, blood pressure, weight
  - Adverse events (NIH criteria)
  - Stool collection or peri-rectal swabs

Participants will be asked if study staff may follow participants passively using their medical records if clinical follow-up and/or stool collection is not viable.

7.4.3 Unscheduled

Occurs at the time of CDI recurrence for participants who experience an infection. Data collection is in addition to early termination visit. Study staff will complete an infection CRF for each CDI. This CRF will be used to track duration of the episode, type, dosage and duration of antibiotics.

- Data points for infectious episode
  - Date
  - Preliminary diagnosis
  - Antibiotic/s
    - Name
    - Formulation (IV, suspension, capsules)
    - Dosage
    - Prescribed duration
    - Final (actual) duration of antibiotics
    - Change in antibiotics regimen
  - Concomitant medication (Name, dose, route of administration, duration)

- o Hospital admission (yes/no)
  - ▪ Name of facility
  - ▪ Department where participant was admitted (ward / step down/ ICU)
  - ▪ Relevant medical procedures
    - Colonoscopy (including preparation)
    - Intestinal surgery
    - Dialysis
    - Other
    - Final diagnosis

7.4.4 **Visit 3** Week 8 (±14 days) after completion of oral vancomycin or placebo

The study physician/nurse/coordinator will perform a clinical assessment and administer a study questionnaire which will specifically inquire about solicited and unsolicited symptoms of AEs. Access to the medical records may be utilized to capture salient changes in healthcare status. Participants will submit a stool sample within each follow-up window.

- Data collection at visit:
  - o Interim medical history with focus on infectious diseases
  - o Concomitant medication
  - o Significant changes in diet
  - o Changes in stool consistency (Bristol Stool Scale) and frequency
  - o Clinical evaluation using standard, structured assessment
  - o Vital signs (if visit performed in-person): Temperature, heart rate, blood pressure, weight
  - o Adverse events (NIH criteria)
  - o Stool collection or peri-rectal swabs

8 Study Analysis

8.1 Sample Size Determination

Power and sample size are based on the main outcome analysis (Aim 1). Based on previous research an absolute decrease of 22% in the proportion of CDI recurrence (h = 0.66) between oral vancomycin versus placebo treatment Hise [5], et. al., 2016) has been shown. Using a 1:1 random allocation ratio, with 80% power and two-tailed p-value < 0.05, we estimated the following sample sizes based on a variety of effect sizes (h) and attrition rates.[49]

Table 4: Sample size estimates

Study	Effect size (h)[a]	Absolute Proportional change	Total Sample Size	Sample size based on 10 % Attrition rate	Sample size based on 20% Attrition rate	Sample size based on 30% Attrition rate
Van Hise	.66	22%	84	94	106	121
	.62	21.5%	100	112	126	144
	.57	20.5%	112	125	141	161
	.53	19.5%	126	141	158	181
	.49	18.5%	140	156	176	201

[a]Effect sizes based on the difference in a (2 *arcsin(SQRT(Pr))) probability transformation (Cohen, 1988).

Using the 1:1 allocation ratio, 80% power for a two-sided p-value < 0.05 will be achieved with sampling between 84 – 144 total, dependent upon the level of attrition. We propose a total sampling of 150 individuals, anticipating no more than a 20% attrition rate, we would be able to detect effects as low as h = 0.57, or a 20.5% decrease in recurrence.

8.2 Statistical Methods

Primary Study Endpoint:

1. Recurrent CDI during therapy and in 8-week period following completion of oral vancomycin therapy.

Secondary Study Endpoints:

1. Changes in biodiversity and function of the gut microbiota between the treatment and comparator group at Visit 3.
2. Changes in gut colonization by vancomycin-resistant enterococcus (VRE) in patients receiving intervention vs placebo group at Visit 3.
3. *C. difficile* positivity on any stool sample as a possible predictor for CDI recurrence within 180 days of intervention vs placebo.

Aim 1

Hypothesis 1a: The proportion of recurrent CDIs at 8 weeks following completion of oral vancomycin therapy will be significantly lower than placebo.

Hypothesis 1b: Time to recurrence of CDI will be significantly longer in the oral vancomycin group than the placebo group.

Initially, basic proportional difference tests for the proportion of CDI recurrence for prophylaxis vancomycin versus placebo for per-protocol and intent-to-treat analysis will be conducted using the Kaplan-Meier method. Multivariate Cox proportional hazard regression will then be used to determine if treatment (vancomycin or placebo) receipt, patient age, and number of previous CDI episodes are predictors of *C.difficile* recurrence. Also, treatment interactions with patient age and prior CDI episodes will be investigated. Finally, time to recurrence by vancomycin group in patients with a prior episode of *C.difficile* infection will be assessed using age-adjusted Cox proportional analysis of the probability of recurrence according to vancomycin group (per-protocol and intent-to-treat analyses).

Aim 2

Hypothesis 2: Alteration of diversity and function of gut microbiota will be greater with oral vancomycin administration.

Multiplex sequencing of amplicons generated from bacterial 16S rRNA genes. 16S rRNA microbiota analysis will be performed for all fecal samples. Genomic DNA will be extracted from feces using a bead-beating protocol, as previously described. [50] We will generate multiplexed barcoded amplicons from the variable region V4 of bacterial 16S rRNA genes, and perform 2x250 bp paired-end sequencing using an Illumina MiSeq, as previously described.[51] Multiplexing in this manner is a cost-effective and efficient approach to document the gut microbiota in humans, as has been demonstrated previously.

Composition and structure of the gut microbiota. Analysis of our 16S rRNA gene libraries will be performed in the analysis software package mothur [52] to determine both the composition and structure of the gut microbiota. Two broad scale analyses, alpha and beta diversity, will be used in order to quantify and compare samples.

For alpha diversity, the number of species present in a sample (richness) will be determined using the Chao1 index [53] and will be coupled to the abundance of the species (evenness) to calculated overall alpha diversity using Shannon's index.[54] Based on our hypotheses, we expect that for our vancomycin group, samples collected from patients prior to vancomycin treatment (visit 1) will be higher in diversity than right after vancomycin

treatment (visit 2), as application of vancomycin will reduce overall diversity. We expect that diversity will increase to levels observed prior to vancomycin treatment (visit 1) for those samples collected 8 weeks after vancomycin treatment (visit 3). For our placebo group, we expect that microbial diversity will remain consistent throughout the trial, and be similar to diversity levels observed for patients in the vancomycin group prior to vancomycin treatment (visit 1). Importantly, given that these patients have recurrent CDI, we also expect that our placebo group will also maintain a higher abundance of *C. difficile* throughout the trial (i.e. lower evenness), and in some cases, may even proliferate to levels consistent with CDI.

Beta diversity analyses will then be used to gain insights into the structure of the gut microbiota and will be performed by first conducting an operational taxonomic unit (OTU) analysis, which defines taxa according to shared sequence similarity, allowing for the identification of specific bacterial members through comparison against the greengenes taxonomic database.[55] Sample comparisons will be performed using a number of metrics, including weighted and unweighted UNIFRAC,[56] and the Bray-Curtis dissimilarity [57] analysis. These approaches quantify the compositional similarity or dissimilarity between microbial communities in terms of both total diversity and phylogenetic distance, and allow for visualization using PCA. We will further identify those specific taxa contributing to the differences between samples by employing correlation analyses on the total community composition via analysis of similarity (ANOSIM)[58] in R[59] and permutation testing of multivariate homogeneity of group dispersions (PERMDISP).[60] Trends causing changes in dispersion will be identified by log-linear models, and those OTUs contributing to the differences between samples, will be identified by an analysis of similarity percentages (SIMPER). [58] We expect that, for the vancomycin treatment group, samples obtained before, during, and after vancomycin treatment will exhibit some commonalities, but likely be different from each other. Application of vancomycin has the effect of “resetting” the microbiota, leading to potentially wholesale colonization by entirely different groups of microbes from the environment (i.e. diet, living conditions, etc.). However, constant dosing of vancomycin at low levels as described in our study may select for a consistent group of microbes able to persist under these conditions (e.g. via formation of biofilms, acquisition of resistance, etc). As a result, we expect that the microbiota composition will be similar between the after vancomycin application (visit 2) and 8 weeks after treatment (visit 3), as this would allow sufficient time for the community to stabilize. An expected outcome would be the overall decrease or loss of *C. difficile* from the community as the stabilized established microbiota is able to resist *C. difficile* invasion, as is known for healthy individuals. For our placebo group, we expect the overall community structure to remain stable throughout the treatment time course, as there is no extreme condition that allows for “resetting” of the microbiota. We expect to see consistent levels of *C. difficile* that may fluctuate in abundance over time, with a concomitant decrease or shift in composition if a CDI occurs due to the proliferation of *C. difficile* outcompeting other members of the established microbiota. Overall, deviations in microbiota are expected to be observed after vancomycin treatment (visit 2) between both groups and continue diverging into 8 weeks after treatment (visit 3). Finally, our correlation analysis will also identify groups of taxa significantly associated with and without vancomycin treatment, and these microbes may represent those capable of outcompeting *C. difficile* (vancomycin treatment) or allow for the proliferation of *C. difficile* (placebo).

Metagenomic analysis. A key goal of our study is to not only determine differences in overall community structure and composition using 16S rRNA sequencing, but to also assess if there are functional differences between the established microbiota. To address this, we will perform shotgun metagenomics on a select number of fecal samples (200) using an Illumina Hi-Seq 2500. We will perform 2x150 bp paired end sequencing and multiplex 12 samples per lane, which will generate an average of 5 Gbp of data per sample. This amount of coverage should be sufficient for analysis, given that antibiotic use for recurrent CDI reduces the overall diversity, relative to healthy individuals. It is expected that the vancomycin treatment group will exhibit even lower diversity than the placebo group, and thus this level of coverage is sufficient for our downstream analysis. All raw sequences from the sequencer passing QC will be assembled using MEGAHIT[61] to generate contigs and used to predict open reading frames (ORFs) using the program prodigal.[62] This library of genes within a given sample will serve as the basis for all downstream analyses using our metagenomics data, as follows. First, all metagenomes will

be phylogenetically binned against the Human Microbiome Project (HMP, http://hmpdacc.org/), which contains over 3,000 genomes of human-associated bacterial strains, including over 800 from the gut alone. By limiting our comparison to the HMP, we will not only reduce the computational time required, as compared to searching against over 20,000+ genomes in the entire GenBank database, but also focus our analyses on only those organisms associated with humans. This comparison will be performed by constructing a custom HMP database for use with the phylogenetic binning program centrifuge. [63] An immediate outcome of this analysis will be to validate our 16S rRNA sequence data, as metagenomics data does not suffer from the bias introduced from PCR, and provide an accurate and absolute measurement of abundance (as opposed to relative abundance estimated via 16S rRNA analysis).

Our generated metagenome data also provides the opportunity to determine if specific functional genes are associated with vancomycin treatment. A key outcome of vancomycin treatment is the establishment of a resident microbiota that may develop the ability to persist repeated application of low-dose vancomycin. This would result in a core group of microbes with potential functional abilities to resist vancomycin application, such as biofilm formation and acquisition of vancomycin resistance genes. We will test this hypothesis using our metagenomics data by assigning and analyzing gene function. To accomplish this, predicted ORF translations from our assembled metagenomes will be subjected to functional annotation using the KEGG orthology (KO) [64], Clusters of Orthologous Groups (COG) [65], Gene Ontology (GO) [66], and Pfam. [67] The use of the multiple annotations will be useful for verifying the predicted function of a given ORF. Comparison between samples for the enrichment of gene sets will be conducted using Fisher's Exact test. Analysis of those gene sets over- and under-enriched between the treatment and placebo group after vancomycin treatment (visit 2) will identify differences attributable to vancomycin use. We predict that genes associated with vancomycin resistance will be enriched in the vancomycin group, relative to the placebo. These would include those genes found in the *vanA-G* operon distributed across numerous bacterial genera including *Bacillus*, *Enterococcus, Staphylococcus,* and *Ruminococcus.*[68] To facilitate this analysis, we will also annotate our predicted ORFs using the Comprehensive Antibiotic Resistance Database (CARD) [68], which specializes in defining antibiotic resistance genes. Genes associated with other mechanisms of antibiotic resistance such as biofilm formation will be determined from our initial annotations, which include numerous well-defined pathways for biofilm formation (e.g. KEGG), especially in Gram-negative organisms that also comprise the microbiota.

Aim 3

Hypothesis 3: The proportion of VRE incidence at 8 weeks following completion of oral vancomycin therapy will be significantly lower than placebo.

Basic proportional difference tests for the proportion of VRE incidence for prophylaxis vancomycin versus placebo for per-protocol and intent-to-treat analysis will be conducted using the Kaplan-Meier method.

Aim 4

Hypothesis 4: *C. difficile* positivity (toxigenic culture) on any stool sample is a predictor for CDI recurrence and vancomycin prophylaxis will decrease risk of recurrence.

Basic proportional difference tests for *C. difficile* recurrence for *C. difficile* asymptomatic positivity versus *C. difficile* negative will be conducted and multivariate analysis will then be used to determine if treatment (vancomycin or placebo) receipt, age, number of previous CDI episodes, asymptomatic *C. difficile* positivity, are predictors of recurrence.

Subject population(s) for analysis will be all-randomized population.

Safety endpoints

Using structured CRFs at scheduled visits as well as weekly phone calls, we will elicit potential adverse effects of oral vancomycin use. These may include nausea, abdominal pain and bloating. The frequency of adverse events will be compared between the active treatment and placebo group. In addition, if VRE infection occurs in a study participant, additional stool samples may be collected as well as the isolate causing the infection to

evaluate if colonization by VRE was present at or before the infection. In addition, we will follow FDA and AHRQ guidance on safety assessment of the intervention.

Subgroup Analysis

Many baseline characteristics are known to be prognostic or suspected confounders for the clinical outcomes in the CDI study population, including age, sex, race/ethnicity, status of hospital stay, and antibiotic use. Internal consistency of the primary efficacy analysis will be assessed in subgroups defined by these covariates. Heterogeneity will be assessed using interaction tests of treatment by these baseline covariates. Any interaction test resulting in a heterogeneity p-value < 0.15 will be further evaluated for clinical plausibility.

8.3 Planned Interim Analysis:

No interim analysis is planned; however, we will rely on the data coordinating center.

9 Data Collection, Handling and Record Keeping

9.1 Data Confidentiality

All members of the investigative team will have completed all human subjects' certification requirements and training in data confidentiality. Research data will be handled with utmost confidentiality and discretion. Subjects will be assigned a unique identification number that can be traced only by the Research Specialist and PI. All subject information will be kept in locked drawers, file cabinets or secure computer files, with access only allowed to research personnel. The study protocol, documentation, data, and all other information generated will be held in strict confidence. No information concerning the study or the data will be released to any unauthorized third party without prior written approval from the sponsor.

The study monitor or other authorized representatives of the sponsor and FDA may inspect all documents and records required to be maintained by the Investigator, including, but not limited to, medical records (office, clinic, or hospital) and pharmacy records for the participants in this study. The clinical study site will permit access to such records.

The extent of the confidentiality of the participants' records must be defined, and participants must be informed that applicable data protection legislation will be followed. Participants must be informed that the monitor(s), auditors(s), IRB and regulatory authority(ies) will be granted direct access to the participant's medical records for verification of clinical trial procedures and/or data without violating the confidentiality of the participant, to the extent permitted by the applicable laws and regulations, and that, by signing a written informed consent form, the participant is authorizing such access. Participants must be informed that records identifying the participant will be kept confidential, and, to the extent permitted by the applicable laws and/or regulations, will not be made publicly available and, if the results of the trial are published, the participant's identity will remain confidential.

9.1.1 Confidentiality of Subject Records

By signing the protocol, the Investigator agrees that the *NIH, IRB, DMC, SMS or Regulatory Agency* representative may consult and/or copy study documents in order to verify CRF data. By signing the consent form, the subject agrees to this process. If study documents will be photocopied during the process of verifying CRF information, the subject will be identified by unique code only and full names and similar identifying information (such as medical record number or social security number) will be masked.

The Clinical Site Investigators will ensure that the identity of subjects will be protected. All study records will be maintained in a secure fashion with access limited to essential study personnel only. All study documents submitted to the Coordinating Center will have identifiers removed other than dates of birth and service and

subjects will be identified with a study-specific identification number only. The Clinical Site Investigators will maintain, in a secure location, an enrollment log that includes subject identifying information and links subjects to their study-specific identification number.

9.2 Data Capture

9.2.1 Source Documents

Each participating site will maintain appropriate medical and research records for this trial, in compliance with ICH E6 GCP, Section 4.9, and regulatory and institutional requirements for the protection of confidentiality of participants. Forms for use as source documents will be developed to mimic the electronic CRFs. Additional source data are all information, original records of clinical findings, observations, or other activities in a clinical trial necessary for the reconstruction and evaluation of the trial. Examples of these original documents and data records include, but are not limited to, hospital records, clinical and office charts, laboratory notes, memoranda, pharmacy dispensing records, recorded data from automated instruments, copies or transcriptions certified after verification as being accurate and complete, microfiches, photographic negatives, microfilm or magnetic media, x-rays, and participant files and records kept at the pharmacy, laboratories, and medico-technical departments involved in the clinical trial.

9.2.2 Case Report Forms

The study case report form (CRF) is a data reporting instrument for the study. All data requested on the CRF must be recorded. All missing data must be explained. All entries should be printed legibly in black ink. If any entry error has been made, to correct such an error, draw a single straight line through the incorrect entry and enter the correct data above it. All such changes must be initialed and dated.

NOTE: If a space on the CRF is left blank because the procedure was not done or the question was not asked, write “N/D”. If the item is not applicable to the individual case, write “N/A”. DO NOT ERASE OR WHITE OUT ERRORS. For clarification of illegible or uncertain entries, print the clarification above the item, then initial and date it.

9.2.2.1 Missing Data

Not all participants of the study will have their stool samples available for analysis, and there will inevitably be missing data. Most methods for handling missing data assume that the data are missing at random, which may not be a valid assumption. Reasons for missing data will be documented and evaluated. If the missing data are extensive, model-based approaches will estimate their effects under various assumptions regarding missing data. For longitudinal data, principal stratification will be used for missing data.[69] Missing data analysis will follow the guideline promulgated in the National Research Council report.[70]

9.2.3 Data Collection Tools

Clinical data (including AEs, concomitant medications, and solicited events data) and clinical laboratory data will be entered into an Internet Data Entry System, REDCap. The data system includes password protection and internal quality checks, such as automatic range checks, to identify data that appear inconsistent, incomplete, or inaccurate. Clinical data will be entered directly from the source documents.

9.3 Data Management

REDCap will be used for electronic data collection and entry at the clinic sites, and all data entered through REDCap will be stored on secure servers provided by study sites. REDCap offers easy data manipulation with audit trails, reporting, monitoring, querying records, and an automated export mechanism to common statistical packages, including R and SAS. It provides a secure, web-based, flexible system with an intuitive interface for sites to enter data and has real time validation rules with automated data type and range checks at the time of data entry. The DCC Project Coordinator will provide real time data review.

9.4 Data and Safety Monitoring

Section 10.6 includes the Data and Safety Monitoring plan as well as a description of the Data Monitoring Committee that will be overseeing this study across all-sites.

9.5 Records Retention

Study files (except for future use consent forms) must be maintained for a minimum of two years after the last approval of a marketing application in an ICH region and until there are no pending or contemplated marketing applications in an ICH region or at least 2 years have elapsed since the formal discontinuation of clinical development of the investigational product. These documents should be retained for a longer period, however, if required by local regulations; for example study data will be retained for a minimum of 7 years following the University of Wisconsin-Madison Policy on Data Stewardship, Access, and Retention. No records will be destroyed without the written consent of the sponsor, if applicable. It is the responsibility of the sponsor to inform the investigator when these documents no longer need to be retained. Consent forms for future use will be maintained as long as the sample exists.

9.6 Specimen Banking

Any leftover stool specimens will be stored and may be used for future research, under a future protocol, to learn more about fecal microbiota. These specimens will be stored indefinitely at the study site repository after the study is completed. In the informed consent document, participants will be given an opportunity to choose whether or not their de-identified barcoded specimens are stored for future use. For participants who choose not to allow storage of their samples for future use, these samples will be destroyed at the end of the study.

There are no benefits to participants in the collection, storage and subsequent research use of specimens. Reports about future research done with participant's samples will NOT be kept in their health records, but participant's samples may be kept with the study records or in other secure areas. Participants can decide if they want their samples to be used for future research or have their samples destroyed at the end of the study. A participant's decision can be changed at any time before the end of the study by notifying the study doctors or nurses in writing. However, if a participant consents to future use and some of their stool has already been used for research purposes, the information from that research may still be used.

Samples may be shared with other investigators at other institutions. Each sample will be encoded (labeled) only with a barcode and a unique tracking number to protect participant's confidentiality.

Research using stored specimens may be conducted by other institutions. Any specimens and data provided to the receiving-institution will be coded. Unequivocally, neither individual personal identifiers nor the key linking coded data to individuals will be released to the receiving-institution.

10 Assessment of Safety

Safety will be assessed by reviewing the frequency and severity of adverse events (AE).

10.1 Specifications of Safety Parameters

Reference safety parameters that are study endpoints (Section 4.1, Study Endpoints).

10.1.1 Definition of Adverse Events (AEs)

Adverse events (AEs) will be recorded at each regular scheduled study visit in the study patient record (source document) as well as on a specific AE case report form (CRF). Given the patient population that will be enrolled, any participant who experiences an event that is unrelated to study participation and is grade 1 or 2 (mild or moderate), will not be recorded as an adverse event. If the event escalates to grade 3 or higher, the event will be recorded.

An AE is any untoward medical occurrence in a study patient or clinical investigation participant administered a pharmaceutical product. An AE does not necessarily have a causal relationship with this treatment. An AE can therefore be any unfavorable and unintended sign (including an abnormal laboratory finding), symptom, or disease temporally associated with the use of a medicinal product, whether or not related to the medicinal product, e.g.:

- any new clinical diagnosis
- any symptom that requires medical clarification or leads to in-patient admission (surgery or accident)
- any suspected adverse drug reaction (ADR)
- any symptom that appears on the study patient's medical records
- any event related in time with the application of the study medication and affecting the health of the study patient (including laboratory value changes)

If there is any doubt as to whether a clinical observation is an AE, the event should be reported. AEs must be graded for severity and relationship to study product. Adverse events of special interest (AESI) will be defined as newly acquired transmissible infectious diseases.

NIH Grading of Severity of the Event

AEs will be assessed by the clinician using the NIH Common Terminology Criteria for Adverse Events (CTCAE), defined grading system. Briefly, the criteria for estimating adverse event severity grade:

- **Grade 1, Mild:** Asymptomatic or mild symptoms; clinical or diagnostic observations only; intervention not indicated.
- **Grade 2, Moderate:** Minimal, local or noninvasive intervention indicated; limiting age-appropriate instrumental activities of daily living (ADL).
- **Grade 3, Severe:** Severe or medically significant but not immediately life-threatening; hospitalization or prolongation of hospitalization indicated; disabling; limiting self-care ADL.
- **Grade 4, Life threatening:** Places the patient or subject at immediate risk of death. It does not include an adverse event that, had it occurred in a more severe form, might have caused death.
- **Grade 5, Death**

10.1.2 Definition of Serious Adverse Events (SAE)

An adverse event or suspected adverse reaction is considered "serious" if, in the view of either the investigator or sponsor, it results in any of the following outcomes:

- Death;
- Life-threatening adverse event*;
- Inpatient hospitalization or prolongation of existing hospitalization;
- A congenital anomaly/birth defect.
- Persistent or significant disability or incapacity or substantial disruption of the ability to conduct normal life function

**Life-threatening adverse event. An adverse event is considered "life-threatening" if, in the view of either the investigator or sponsor, its occurrence places the patient or participant at immediate risk of death. It does not include an adverse event which, had it occurred in a more severe form, might have caused death.*

Any adverse event or suspected adverse reaction that meets the criteria for serious adverse event will be:

- Recorded on the appropriate SAE CRF
- Followed through resolution by a study physician
- Reviewed and evaluated by a study clinician
- Reported to the appropriate entities, including the sponsor, DMC and IRB, if applicable

10.1.3 Definition of Unanticipated Problems (UP)

OHRP considers unanticipated problems involving risks to participants or others to include, in general, any incident, experience, or outcome that meets **all** of the following criteria:

- Unexpected in terms of nature, severity, or frequency given (a) the research procedures that are described in the protocol-related documents, such as the IRB-approved research protocol and informed consent document; and (b) the characteristics of the participant population being studied;
- Related or possibly related to participation in the research ("possibly related" means there is a reasonable possibility that the incident, experience, or outcome may have been caused by the procedures involved in the research); and
- Suggests that the research places participants or others at a greater risk of harm (including physical, psychological, economic, or social harm) than was previously known or recognized.

An incident, experience, or outcome that meets the definition of an UP generally will warrant consideration of changes to the protocol or consent in order to protect the safety, welfare, or rights of participants or others. Other UPs may warrant corrective actions at a specific study site. Examples of corrective actions or changes that might need to be considered in response to an UP include:

- Modification of inclusion or exclusion criteria to mitigate the newly identified risks
- Implementation of additional safety monitoring procedures
- Suspension of enrollment of new participants or halting of study procedures for enrolled participants
- Modification of informed consent documents to include a description of newly recognized risks
- Provision of additional information about newly recognized risks to previously enrolled participants.

10.2 Classification of an Adverse Event

10.2.1 Severity of Event

All AEs will be assessed by the clinician using the CTCAE Refer to the NIH grading of severity of the event guidance listed in section 10.1.1 of this protocol.

10.2.2 Relationship to Study Agent

For all collected AEs, the clinician who examines and evaluates the participant will determine the AE's causality based on temporal relationship and his/her clinical judgment. The degree of certainty about causality will be graded using the categories below.

Definitely Related – There is clear evidence to suggest a causal relationship, and other possible contributing factors can be ruled out. The clinical event, including an abnormal laboratory test result, occurs in a plausible time relationship to drug administration and cannot be explained by concurrent disease or other drugs or chemicals. The response to withdrawal of the drug (de-challenge) should be clinically plausible. The event must be pharmacologically or phenomenologically definitive, with use of a satisfactory re-challenge procedure if necessary.
Probably Related – There is evidence to suggest a causal relationship, and the influence of other factors is unlikely. The clinical event, including an abnormal laboratory test result, occurs within a reasonable time after

administration of the drug, is unlikely to be attributed to concurrent disease or other drugs or chemicals, and follows a clinically reasonable response on withdrawal (de-challenge). Re-challenge information is not required to fulfill this definition.
Possibly Related – There is some evidence to suggest a causal relationship (e.g., the event occurred within a reasonable time after administration of the trial medication). However, other factors may have contributed to the event (e.g., the participant's clinical condition, other concomitant events). Although an AE may rate only as "possibly related" soon after discovery, it can be flagged as requiring more information and later be upgraded to "probably related" or "definitely related," as appropriate.
Unlikely related – A clinical event, including an abnormal laboratory test result, whose temporal relationship to drug administration makes a causal relationship improbable (e.g., the event did not occur within a reasonable time after administration of the trial medication) and in which other drugs or chemicals or underlying disease provides plausible explanations (e.g., the participant's clinical condition, other concomitant treatments).
Not Related – The AE is completely independent of study drug administration, and/or evidence exists that the event is definitely related to another etiology. There must be an alternative, definitive etiology documented by the clinician.

10.2.3 Expectedness

PI will be responsible for determining whether an AE is expected or unexpected. An AE will be considered unexpected if the nature, severity, or frequency of the event is not consistent with the risk information previously described for the study agent.

GUIDANCE: An AE or suspected adverse reaction is considered "unexpected" if it is not listed in the IB or is not listed at the specificity or severity that has been observed; or, if an IB is not required or available, is not consistent with the risk information described in the general investigational plan or elsewhere in the protocol, as amended. For example, under this definition, hepatic necrosis would be unexpected (by virtue of greater severity) if the IB referred only to elevated hepatic enzymes or hepatitis. Similarly, cerebral thromboembolism and cerebral vasculitis would be unexpected (by virtue of greater specificity) if the IB listed only cerebral vascular accidents. "Unexpected," as used in this definition, also refers to AEs or suspected adverse reactions that are mentioned in the IB as occurring with a class of drugs or as anticipated from the pharmacological properties of the drug, but are not specifically mentioned as occurring with the particular drug under investigation.

10.3 Time period and frequency for event assessment and follow-up

The occurrence of an AE or SAE may come to the attention of study personnel during study visits and interviews of a study participant presenting for study visits or procedures, or upon review by a study monitor. All AEs including local and systemic reactions not meeting the criteria for SAEs will be captured on the appropriate CRF. Information to be collected includes event description, clinician's assessment of severity, relationship to study product (assessed only by those with the training and authority to make a diagnosis), and time of resolution/stabilization of the event. All AEs occurring while on study must be documented appropriately regardless of relationship. All AEs will be followed to adequate resolution.

Any medical condition that is present at the time that the participant is screened will be considered as baseline and not reported as an AE. However, if the study participant's condition deteriorates at any time during the study, it will be recorded as an AE. UPs will be recorded in the data collection system throughout the study.
Changes in the severity of an AE will be documented to allow an assessment of the duration of the event at each level of severity to be performed. AEs characterized as intermittent require documentation of onset and duration of each episode.

The PI will record all reportable events with start dates occurring any time after informed consent is obtained until the last day of study participation. At each study visit, the investigator will inquire about the occurrence of

AE/SAEs since the last visit. Events will be followed for outcome information until resolution or stabilization, even if they continue after study completion.

10.4 Reporting procedures

10.4.1 Adverse Event Reporting

Study participants will be instructed to contact the study team or physician if any serious or unexpected adverse event occurs. Study staff will inquire about AEs at each study visit. Reported AE's will be recorded in detail in an AE CRF.

AE information to be collected in the AE CRF:

- Nature/description of the event
- Start date
- End Date
- Severity
- Seriousness
- Expectedness
- Attribution/relatedness to the study drug, patient population, and study procedures
- Outcome
- Treatment (i.e. how and if the AE was treated with medications or non-medication treatment). *Treatment with medications will documented on the concomitant medication log.*
- Action taken with regards to study intervention (options include: None, Interrupted, Discontinued, Not Applicable)

Measures at the onset of adverse events are classified and described as follows:

- None, i.e. the study medication was not changed
- The study medication was withdrawn and/or
- Other measures

The course and outcome of the adverse event will be commented on as follows:

- Recovered without sequelae
- Not yet recovered
- Recovered with sequelae
- Fatal

10.4.1.1 Serious adverse event reporting

The study clinician will complete a SAE Form within the following timelines:

- All SAEs that result in death or are immediately life-threatening events related (possibly, probably or definitely) to the study drug and are considered Unexpected will be recorded on the SAE Form and submitted to the DCC, study sponsor, DMC and IRB (if applicable) within 24 hours of site awareness. See Section 1, Key Roles, for contact information.
- Other SAEs regardless of relationship will be submitted to the DCC, study sponsor and DMC (if applicable) within 72 hours of site awareness. These events may need to be submitted to the local IRB if the event meets the reporting requirements, consistent with their defined timelines.

All SAEs will be followed until satisfactory resolution or until the site investigator deems the event to be chronic or the adherence to be stable. Other supporting documentation of the event may be requested by the DCC,

DMC, SMS or other regulatory authority(ies) and should be provided as soon as possible. The study sponsor will be responsible for notifying FDA of any unexpected fatal or life-threatening suspected adverse reaction as soon as possible but in no case later than 7 calendar days after the sponsor's initial receipt of the information.

10.4.1.2 Unanticipated problem reporting

The site will be responsible for completing and submitting their IRB's Unanticipated Problem (UP) report form. Incidents that meet the OHRP criteria for UPs will be reported promptly on the following timeline:

- UPs that are SAEs will be reported to the IRB and to the DCC 24 to 72 hours (depending on the nature of the SAE as described above in 10.4.1.1) of the investigator becoming aware of the event.
- Any other UP will be reported to the IRB and to the DCC within 14 business days of the investigator becoming aware of the problem.

The UP report will include the following information:

- Protocol identifying information: protocol title and number, PI's name, and the IRB project number;
- A detailed description of the event, incident, experience, or outcome;
- An explanation of the basis for determining that the event, incident, experience, or outcome represents an UP;
- A description of any changes to the protocol or other corrective actions that have been taken or are proposed in response to the UP.

10.4.1.3 Events of special interest

Not-applicable.

10.4.1.4 Reporting of pregnancy

Not-applicable.

10.5 Study Halting Rules

Study enrollment halting rules

Enrollment in the study will be suspended for conduct of a safety review by the DMC:

- Three or more of the randomized participants in a study treatment group have a Grade 3 AE of the same organ system deemed related to the study intervention.
- Death of an enrolled participant definitely related to the study intervention.
- An overall pattern of symptomatic, clinical, or laboratory events that the Medical Monitor considers related to study product and that may appear minor in terms of individual events, but that may collectively represent a serious potential concern for safety.

Individual's halting rules

Participants who meet any of the following criteria must be assessed by the PI to determine if it is in the participant's best interest to stop the study product(s):

- Participant choice (withdrawal of consent)
- Participant's non-compliance.
- Development of a significant medical condition and/or participation in the study is no longer in the best interest of the participant.

10.6 Safety Oversight

Data and Safety Monitoring Board (DSMB): Safety oversight will be under the direction of the ICTR Data Monitoring Committee (DMC). The UW ICTR DMC is comprised of experienced members (core plus ad hoc) with expertise required to oversee this study. The DMC members will review protocol-specific reports created by statisticians using data pulled from the Research Electronic Data Capture (REDCap) data management tool. These standard reports will include an overview of study objectives, a review of actual and projected accrual rates, an evaluation of patient demographics for balance of randomization, and a summary of the number and seriousness of adverse events. An interim analysis of study results may be performed and source documents may be reviewed to allow the DMC to independently judge whether the overall integrity and conduct of the protocol remain acceptable based on data provided and reported by the Principal Investigator. The DMC will make recommendations to the Principal Investigator that could include actions of continuation, modification, suspension, or termination.

In providing oversight for the conduct of this study, the ICTR DMC will meet every 12 months during the 5-year study to review all adverse events. Additional meetings may be scheduled as determined by the DMC or as requested by the PI. The predefined stopping points for this study will include the boundaries for excess harm and excess rates as defined in study halting rules, or an observed excess harm which in the judgment of the DMC, is excessive. We will submit all reportable events to the DMC and the Health Sciences IRB in accordance with their reporting guidelines.

10.7 Unblinding Procedure

Unblinding will be done in emergent circumstances where the identity of the study medication needs to be known. All efforts will be made to maintain blinding except in the case of urgent medical necessity. If a subject needs to be unblinded, the study staff should contact the medical monitor prior to any unblinding procedures.

11 Study Monitoring, Auditing, and Inspecting

While many institutions involved in clinical research conduct various types of quality assurance reviews and audits, University of Wisconsin Institute for Clinical and Translational Research (ICTR) is one of a few institutions to offer independent Study Monitoring Services, a robust academic equivalent to the industry Contract Research Organization (CRO) standards for ongoing study monitoring.

Dr. Nasia Safdar, the sponsor-investigator of this study, has contracted with ICTR Study Monitoring Service (SMS) to provide ongoing monitoring throughout the life cycle of the study. Refer to the Study Monitoring Plan.

Three (3) study sites will be enrolling subjects into this study. Monitoring will be conducted at all three sites to ensure that the rights and well-being of human subjects are protected, that the reported trial data are accurate, complete, and verifiable, and that the conduct of the trial is in compliance with the currently approved protocol/amendment(s), with GCP, and with applicable regulatory requirement(s). Monitoring refers to the methods used by sponsors or sponsor-investigators of investigational studies, or Contract Research Organizations (CROs) delegated site monitoring responsibilities, to oversee the conduct of, and reporting of data from, clinical investigations. Site monitoring includes ensuring appropriate clinical investigator supervision of study site staff and third party contractors. Monitoring activities include communication with the clinical investigator and study site staff; and protocol review.

For this study, UW ICTR SMS personnel will conduct a Site Initiation Visit (SIV) and ongoing Interim Monitoring Visits (IMVs), either on-site, remotely, centrally or a combination thereof, throughout the duration of the study.

During IMVs, the monitors will review study materials, including but not limited to: regulatory files, consent forms, case report forms, and drug accountability logs.

Monitoring will consist of review of all (100%) of the study-related subject research records representing 10-15% of those enrolled. SMS personnel could increase the percentage of study or subject records to be reviewed if warranted by the ongoing monitoring findings, resulting in a partial or full review of up to 100% of the study-related subject records. Based on the plan to review approximately 10-15% of the subject records, it is anticipated that the following monitoring visit schedule will implemented; 2 SIVs (for 3 sites), 3 Initial IMVs (1 at each site) following the enrollment of the first subject(s), followed by approximately 2 additional IMVs at each site (total of 6) through the end of the study. The frequency of these visits will based on the subject enrollment rate. Three (3) Close-Out Visits (COVs) will be conducted, one at each site.

The study monitor(s) will work closely with the ICTR DMC statistician, the study statistician, the DCC and the CCC.

11.1 Medical Monitoring

Dr. Nasia Safdar is the medical monitor for this study. It is the responsibility of the Principal Investigator to oversee the safety of the study at his/her site. Medical monitoring will include a regular assessment of the number and type of serious adverse events as well as provide guidance to all sites regarding the protocol.

11.2 Protocol Deviations

A protocol deviation is any noncompliance with the clinical trial protocol, GCP, or MOP requirements. The noncompliance may be either on the part of the participant, the investigator, or the study site staff. As a result of deviations, corrective actions are to be developed by the site and implemented promptly.
These practices are consistent with ICH E6:

- 4.5 Compliance with Protocol, sections 4.5.1, 4.5.2, and 4.5.3
- 5.1 Quality Assurance and Quality Control, section 5.1.1
- 5.20 Noncompliance, sections 5.20.1, and 5.20.2.

It is the responsibility of the site PI/study staff to use continuous vigilance to identify and report deviations within five working days of identification of the protocol deviation, or within five working days of the scheduled protocol-required activity. All deviations must be promptly reported to the study PI.

All protocol deviations, as defined above, must be addressed in study participant source documents. A completed copy of the Protocol Deviation Form must be maintained in the Regulatory File, as well as in the participant's source document. Protocol deviations must be sent to the local IRB/IEC per their guidelines. The site PI/study staff is responsible for knowing and adhering to their IRB requirements.

11.3 Auditing and Inspecting

The investigator will permit study-related monitoring, audits, and inspections by the EC/IRB (or their representatives), the sponsor, government regulatory bodies, of all study related documents (e.g. source documents, regulatory documents, data collection instruments, study data etc.). The investigator will ensure the capability for inspections of applicable study-related facilities (e.g. pharmacy, diagnostic laboratory, etc.).

11.4 Subject Compliance Monitoring

Subjects will be provided a dose diary to record daily administration of vancomycin/placebo capsules. Vancomycin or placebo capsules will be consumed under direct supervision of study or clinical staff as appropriate while in an inpatient setting.

12 Ethical Considerations

This study is to be conducted according to US and international standards of Good Clinical Practice (FDA Title 21 part 312 and International Conference on Harmonization guidelines), applicable government regulations, applicable local and state laws, and Institutional research policies and procedures.

This protocol and any amendments will be submitted to a properly constituted independent Ethics Committee (EC) or Institutional Review Board (IRB), in agreement with local legal prescriptions, for formal approval of the study conduct. The decision of the EC/IRB concerning the conduct of the study will be made in writing to the investigator and a copy of this decision will be provided to the sponsor before commencement of this study. The investigator should provide a list of EC/IRB members and their affiliate to the sponsor.

All subjects for this study will be provided a consent form describing this study and providing sufficient information for subjects to make an informed decision about their participation in this study. See Attachment for a copy of the Subject Informed Consent Form. This consent form will be submitted with the protocol for review and approval by the EC/IRB for the study. The formal consent of a subject, using the EC/IRB-approved consent form, must be obtained before that subject undergoes any study procedure. The consent form must be signed by the subject or legally acceptable surrogate, and the investigator-designated research professional obtaining the consent.

13 Study Finances

13.1 Funding Source

This study is financed through a grant from the Agency for Healthcare Research and Quality (AHRQ).

13.2 Conflict of Interest

Any investigator who has a conflict of interest with this study (patent ownership, royalties, or financial gain greater than the minimum allowable by their institution, etc.) must have the conflict reviewed by a properly constituted Conflict of Interest Committee with a Committee-sanctioned conflict management plan that has been reviewed and approved by the study sponsor prior to participation in this study. All investigators will follow the study site conflict of interest policy.

14 Publication Plan

Publications information will be included in periodic reports sent to the sponsor.

15 References

1. Pacheco SM, Johnson S. Important clinical advances in the understanding of Clostridium difficile infection. *Current opinion in gastroenterology.* Jan 2013;29(1):42-48.

2. Evans CT, Safdar N. Current Trends in the Epidemiology and Outcomes of Clostridium difficile Infection. *Clin. Infect. Dis.* May 15 2015;60 Suppl 2:S66-71.

3. Lessa FC, Mu Y, Bamberg WM, et al. Burden of Clostridium difficile Infection in the United States. *New England Journal of Medicine.* 2015;372(9):825-834.

4. Nanwa N, Kendzerska T, Krahn M, et al. The economic impact of Clostridium difficile infection: a systematic review. *Am. J. Gastroenterol.* Apr 2015;110(4):511-519.
5. Kelly CP, LaMont JT. Clostridium difficile--more difficult than ever. *The New England journal of medicine.* Oct 30 2008;359(18):1932-1940.
6. O'Horo JC, Jindai K, Kunzer B, Safdar N. Treatment of recurrent Clostridium difficile infection: a systematic review. *Infection.* Feb 2014;42(1):43-59.
7. Zellmer C, De Wolfe TJ, Van Hoof S, Blakney R, Safdar N. Patient Perspectives on Fecal Microbiota Transplantation for Clostridium Difficile Infection. *Infectious diseases and therapy.* Apr 5 2016.
8. Garey KW, Aitken SL, Gschwind L, et al. Development and Validation of a Clostridium difficile Health-related Quality-of-Life Questionnaire. *J. Clin. Gastroenterol.* Jan 19 2016.
9. van Nood E, Vrieze A, Nieuwdorp M, et al. Duodenal infusion of donor feces for recurrent Clostridium difficile. *N. Engl. J. Med.* Jan 31 2013;368(5):407-415.
10. Goldberg EJ, Bhalodia S, Jacob S, et al. Clostridium difficile infection: A brief update on emerging therapies. *American journal of health-system pharmacy : AJHP : official journal of the American Society of Health-System Pharmacists.* Jun 15 2015;72(12):1007-1012.
11. Tran MC, Claros MC, Goldstein EJ. Therapy of Clostridium difficile infection: perspectives on a changing paradigm. *Expert opinion on pharmacotherapy.* Dec 2013;14(17):2375-2386.
12. Carignan A, Poulin S, Martin P, et al. Efficacy of Secondary Prophylaxis With Vancomycin for Preventing Recurrent Clostridium difficile Infections. *Am J Gastroenterol.* Dec 2016;111(12):1834-1840.
13. Van Hise NW, Bryant AM, Hennessey EK, Crannage AJ, Khoury JA, Manian FA. Efficacy of Oral Vancomycin in Preventing Recurrent Clostridium difficile Infection in Patients Treated With Systemic Antimicrobial Agents. *Clin. Infect. Dis.* Sep 1 2016;63(5):651-653.
14. Almyroudis NG, Lesse AJ, Hahn T, et al. Molecular epidemiology and risk factors for colonization by vancomycin-resistant Enterococcus in patients with hematologic malignancies. *Infect. Control Hosp. Epidemiol.* May 2011;32(5):490-496.
15. Banach DB, Peaper DR, Fortune BE, Emre S, Dembry LM. The clinical and molecular epidemiology of pre-transplant vancomycin-resistant enterococci colonization among liver transplant recipients. *Clinical transplantation.* Mar 2016;30(3):306-311.
16. de Bruin MA, Riley LW. Does vancomycin prescribing intervention affect vancomycin-resistant enterococcus infection and colonization in hospitals? A systematic review. *BMC infectious diseases.* 2007;7:24.
17. Fridkin SK, Edwards JR, Courval JM, et al. The effect of vancomycin and third-generation cephalosporins on prevalence of vancomycin-resistant enterococci in 126 U.S. adult intensive care units. *Annals of internal medicine.* Aug 7 2001;135(3):175-183.
18. Musher DM, Aslam S, Logan N, et al. Relatively poor outcome after treatment of Clostridium difficile colitis with metronidazole. *Clin. Infect. Dis.* Jun 1 2005;40(11):1586-1590.
19. Pepin J, Alary ME, Valiquette L, et al. Increasing risk of relapse after treatment of Clostridium difficile colitis in Quebec, Canada. *Clin Infect Dis.* Jun 2005;40(11):1591-1597.
20. Lee CH, Steiner T, Petrof EO, et al. Frozen vs Fresh Fecal Microbiota Transplantation and Clinical Resolution of Diarrhea in Patients With Recurrent Clostridium difficile Infection: A Randomized Clinical Trial. *JAMA.* Jan 2016;315(2):142-149.
21. Weiner LM, Fridkin SK, Aponte-Torres Z, et al. Vital Signs: Preventing Antibiotic-Resistant Infections in Hospitals - United States, 2014. *MMWR. Morb. Mortal. Wkly. Rep.* 2016;65(9):235-241.
22. Hunter JC, Mu Y, Dumyati GK, et al. Burden of Nursing Home-Onset Clostridium difficile Infection in the United States: Estimates of Incidence and Patient Outcomes. *Open forum infectious diseases.* Jan 2016;3(1):ofv196.
23. Zimlichman E, Henderson D, Tamir O, et al. Health care-associated infections: a meta-analysis of costs and financial impact on the US health care system. *JAMA internal medicine.* Dec 9-23 2013;173(22):2039-2046.
24. Madeo M, Boyack M. Using the lived experiences of patients with Clostridium difficile infection to improve care. *Nurs. Times.* Sep 14-20 2010;106(36):10-13.

25. McFarland LV, Surawicz CM, Rubin M, Fekety R, Elmer GW, Greenberg RN. Recurrent Clostridium difficile disease: epidemiology and clinical characteristics. *Infect. Control Hosp. Epidemiol.* Jan 1999;20(1):43-50.
26. Pakyz AL, Moczygemba LR, VanderWielen LM, Edmond MB. Fecal microbiota transplantation for recurrent Clostridium difficile infection: The patient experience. *Am. J. Infect. Control.* May 1 2016;44(5):554-559.
27. Becerra MB, Becerra BJ, Banta JE, Safdar N. Impact of Clostridium difficile infection among pneumonia and urinary tract infection hospitalizations: an analysis of the Nationwide Inpatient Sample. *BMC infectious diseases.* 2015;15:254.
28. Burke KE, Lamont JT. Fecal transplantation for recurrent Clostridium difficile infection in older adults: a review. *J. Am. Geriatr. Soc.* Aug 2013;61(8):1394-1398.
29. Kim HH, Kim YS, Han DS, et al. Clinical differences in Clostridium difficile infection based on age: a multicenter study. *Scand. J. Infect. Dis.* Jan 2014;46(1):46-51.
30. Louie TJ, Miller MA, Crook DW, et al. Effect of age on treatment outcomes in Clostridium difficile infection. *J. Am. Geriatr. Soc.* Feb 2013;61(2):222-230.
31. Rao K, Micic D, Chenoweth E, et al. Poor functional status as a risk factor for severe Clostridium difficile infection in hospitalized older adults. *J. Am. Geriatr. Soc.* Oct 2013;61(10):1738-1742.
32. Hopkins MJ, Macfarlane GT. Changes in predominant bacterial populations in human faeces with age and with Clostridium difficile infection. *J. Med. Microbiol.* May 2002;51(5):448-454.
33. Tart SB. The role of vancomycin and metronidazole for the treatment of Clostridium difficile-associated diarrhea. *Journal of pharmacy practice.* Oct 2013;26(5):488-490.
34. Surawicz CM. Clostridium difficile infection: risk factors, diagnosis and management. *Current treatment options in gastroenterology.* Mar 2015;13(1):121-129.
35. Bagdasarian N, Rao K, Malani PN. Diagnosis and treatment of Clostridium difficile in adults: a systematic review. *JAMA.* Jan 2015;313(4):398-408.
36. Vrieze A, Out C, Fuentes S, et al. Impact of oral vancomycin on gut microbiota, bile acid metabolism, and insulin sensitivity. *J Hepatol.* Apr 2014;60(4):824-831.
37. Lewis BB, Buffie CG, Carter RA, et al. Loss of Microbiota-Mediated Colonization Resistance to Clostridium difficile Infection With Oral Vancomycin Compared With Metronidazole. *J. Infect. Dis.* Nov 15 2015;212(10):1656-1665.
38. CDC. A*ntibiotic Resistance Threats in the United States, 2013.*(CDC, 2013).
39. Song X, Srinivasan A, Plaut D, Perl TM. Effect of nosocomial vancomycin-resistant enterococcal bacteremia on mortality, length of stay, and costs. *Infect Control Hosp Epidemiol.* Apr 2003;24(4):251-256.
40. Pelz RK, Lipsett PA, Swoboda SM, et al. Vancomycin-sensitive and vancomycin-resistant enterococcal infections in the ICU: attributable costs and outcomes. *Intensive Care Med.* Jun 2002;28(6):692-697.
41. Ziakas PD, Thapa R, Rice LB, Mylonakis E. Trends and significance of VRE colonization in the ICU: a meta-analysis of published studies. *PLoS One.* 2013;8(9):e75658.
42. Hendrix CW, Hammond JM, Swoboda SM, et al. Surveillance strategies and impact of vancomycin-resistant enterococcal colonization and infection in critically ill patients. *Ann Surg.* Feb 2001;233(2):259-265.
43. Jung E, Byun S, Lee H, Moon SY. Vancomycin-resistant Enterococcus colonization in the intensive care unit: clinical outcomes and attributable costs of hospitalization. *Am J Infect Control.* Oct 2014;42(10):1062-1066.
44. Diet History Questionnaire, Version 2.0. National Institutes of Health, Epidemiology and Genomics Research Program, National Cancer Institute. 2010.
45. Heaton KW, O'Donnell LJ. An office guide to whole-gut transit time. Patients' recollection of their stool form. *J Clin Gastroenterol.* Jul 1994;19(1):28-30.
46. Lewis SJ, Heaton KW. Stool form scale as a useful guide to intestinal transit time. *Scand J Gastroenterol.* Sep 1997;32(9):920-924.

47. Aagaard K, Petrosino J, Keitel W, et al. The Human Microbiome Project strategy for comprehensive sampling of the human microbiome and why it matters. *FASEB journal : official publication of the Federation of American Societies for Experimental Biology.* Mar 2013;27(3):1012-1022.
48. Muldoon EG, Epstein L, Logvinenko T, Murray S, Doron SI, Snydman DR. The impact of cefepime as first line therapy for neutropenic fever on Clostridium difficile rates among hematology and oncology patients. *Anaerobe.* Dec 2013;24:79-81
49. Cohen J. Statistical power analysis for the behavioral sciences (Rev. ed.). New York: Academic Press,1977.
50. Faith JJ, Rey FE, O'Donnell D, et al. Creating and characterizing communities of human gut microbes in gnotobiotic mice. *ISME J.* Sep 2010;4(9):1094-1098.
51. Kozich JJ, Westcott SL, Baxter NT, Highlander SK, Schloss PD. Development of a dual-index sequencing strategy and curation pipeline for analyzing amplicon sequence data on the MiSeq Illumina sequencing platform. *Appl Environ Microbiol.* Sep 2013;79(17):5112-5120.
52. Schloss PD, Westcott SL, Ryabin T, et al. Introducing mothur: open-source, platform-independent, community-supported software for describing and comparing microbial communities. *Applied and environmental microbiology.* Dec 2009;75(23):7537-7541.
53. Chao A. Non-parametric estimation of the number of classes in a population. Scandinavian Journal of Statistics, 11:265-270. . 1984.
54. Shannon CE. A mathematical theory of communication. Bell System Technical Journal, 27: 379–423. 1948.
55. DeSantis TZ, P. Hugenholtz, N. Larsen, et al. Greengenes, a Chimera-Checked 16S rRNA Gene Database and Workbench Compatible with ARB. Appl Environ Microbiol 72:5069-72. 2006.
56. Lozupone C, Lladser ME, Knights D, Stombaugh J, Knight R. UniFrac: an effective distance metric for microbial community comparison. *The ISME journal.* Feb 2011;5(2):169-172.
57. Bray JR, Curtis JT. An ordination of upland forest communities of southern Wisconsin. Ecological Monographs 27:325-349. 1957.
58. Clarke KR. "Non-parametric multivariate analyses of changes in community structure". Austral Ecology. 18 (1): 117–143. 1993.
59. Team RC. R: A language and environment for statistical computing, R Foundation for Statistical Computing, Vienna, Austria. http://www.R-project.org/. 2015.
60. Anderson MJ, Ellingsen KE, McArdle BH. Multivariate dispersion as a measure of beta diversity. *Ecol Lett.* Jun 2006;9(6):683-693.
61. Li D, Liu CM, Luo R, Sadakane K, Lam TW. MEGAHIT: an ultra-fast single-node solution for large and complex metagenomics assembly via succinct de Bruijn graph. *Bioinformatics.* May 2015;31(10):1674-1676.
62. Hyatt D, Chen GL, Locascio PF, Land ML, Larimer FW, Hauser LJ. Prodigal: prokaryotic gene recognition and translation initiation site identification. *BMC Bioinformatics.* Mar 2010;11:119.
63. Kim D, Song L, Breitwieser FP, SL. S. Centrifuge: rapid and sensitive classification of metagenomic sequences. October 17, 2016. Genome Research doi: 10.1101/gr.210641.116. 2016.
64. Moriya Y,Itoh M, Okuda S, Yoshizawa AC, Kanehisa M. KAAS: an automatic genome annotation and pathway reconstruction server. *Nucleic Acids Res.* Jul 2007;35(Web Server issue):W182-185.
65. Galperin MY, Makarova KS, Wolf YI, EV. K. Expanded microbial genome coverage and improved protein family annotation in the COG database. Nucleic acids research. 43(Database issue):D261-9. 2015.
66. Ashburner M, Ball CA, Blake JA, et al. Gene ontology: tool for the unification of biology. The Gene Ontology Consortium. *Nat Genet.* May 2000;25(1):25-29.
67. Finn RD, Tate J, Mistry J, et al. The Pfam protein families database. Nucleic Acids Res. 36 (Database issue): D281–8. 2008.
68. McArthur AG, Waglechner N, Nizam F, et al. The comprehensive antibiotic resistance database. *Antimicrob Agents Chemother.* Jul 2013;57(7):3348-3357.

69. Frangakis CE, Rubin DB. Principal stratification in causal inference. *Biometrics.* 2002;58(1):21-29

70. Council. NR. The Prevention and Treatment of Missing Data in Clinical Trials.; 2010; Washington, DC:.
