# Supplementary material for: Oral Vancomycin for Prevention of Recurrent Clostridioides difficile Infection: A Randomized Clinical Trial
Source: JAMA Netw Open. 2025 Jul 2;8(7):e2517834. doi: 10.1001/jamanetworkopen.2025.17834 (PMC12223870; doi:10.1001/jamanetworkopen.2025.17834)
Supplement: Supplement 2. — eAppendix 1. Secondary As-Completed-Treatment Analyses Results eTable 1. As-Completed-Treatment Analysis of Primary and Secondary Outcomes eFigure 1. Estimated Nonrecurrence Probability Over Time Between Treatment Groups for the As-Completed-Treatment Population eAppendix 2. Comparison of Primary Outcome, Stratified by Study Sites eFigure 2. Estimated Nonrecurrence Probability Over Time Between Treatment Groups for the As-Randomized Population, Stratified by Study Sites eTable 2. Comparison of Recurrence Rates Between Intervention Group and Placebo Group at 10-Day Intervals After the Intervention, Stratified by Study Sites eAppendix 3. Primary and Secondary Outcomes in Participants Taking Higher-Risk Antibiotics eTable 3. Primary and Secondary Outcomes in Participants Taking High-Risk Antibiotics eFigure 3. Estimated Nonrecurrence Probability Over Time Between Treatment Groups for the Randomized Participants Taking High-Risk Antibiotics eAppendix 4. Primary and Secondary Outcomes in Immunocompromised Participants eTable 4. Primary and Secondary Outcomes in Immunocompromised Participants eFigure 4. Estimated Nonrecurrence Probability Over Time Between Treatment Groups for the Randomized Participants With an Immunocompromising Condition eAppendix 5. Primary and Secondary Outcomes in Participants Aged 65 Years or Older eTable 5. Primary and Secondary Outcomes in Participants Aged 65 Years or Older eFigure 5. Estimated Nonrecurrence Probability Over Time Between Treatment Groups for the Randomized Participants Aged 65 Years or Older [file jamanetwopen-e2517834-s002.pdf]

Supplementary Online Content

Keating JA, Xu T, Graham MB, et al. Oral vancomycin for prevention of recurrent *Clostridioides difficile* infection: a randomized clinical trial. *JAMA Netw Open*. 2025;8(7):e2517834. doi:10.1001/jamanetworkopen.2025.17834

eAppendix 1. Secondary As-Completed-Treatment Analyses Results

eTable 1. As-Completed-Treatment Analysis of Primary and Secondary Outcomes

eFigure 1. Estimated Nonrecurrence Probability Over Time Between Treatment Groups for the As-Completed-Treatment Population

eAppendix 2. Comparison of Primary Outcome, Stratified by Study Sites

eFigure 2. Estimated Nonrecurrence Probability Over Time Between Treatment Groups for the As-Randomized Population, Stratified by Study Sites

eTable 2. Comparison of Recurrence Rates Between Intervention Group and Placebo Group at 10-Day Intervals After the Intervention, Stratified by Study Sites

eAppendix 3. Primary and Secondary Outcomes in Participants Taking Higher-Risk Antibiotics

eTable 3. Primary and Secondary Outcomes in Participants Taking High-Risk Antibiotics

eFigure 3. Estimated Nonrecurrence Probability Over Time Between Treatment Groups for the Randomized Participants Taking High-Risk Antibiotics

eAppendix 4. Primary and Secondary Outcomes in Immunocompromised Participants

eTable 4. Primary and Secondary Outcomes in Immunocompromised Participants

eFigure 4. Estimated Nonrecurrence Probability Over Time Between Treatment Groups for the Randomized Participants With an Immunocompromising Condition

eAppendix 5. Primary and Secondary Outcomes in Participants Aged 65 Years or Older

eTable 5. Primary and Secondary Outcomes in Participants Aged 65 Years or Older

eFigure 5. Estimated Nonrecurrence Probability Over Time Between Treatment Groups for the Randomized Participants Aged 65 Years or Older

This supplementary material has been provided by the authors to give readers additional information about their work.

© 2025 Keating JA et al. *JAMA Network Open*.

eAppendix 1. Secondary As-Completed-Treatment Analyses Results

Out of 60 patients who completed all three visits during the follow-up, 15/31 patients in the oral vancomycin group and 19/29 patients in the placebo group experienced a rCDI (p=0.18; Table S1). A log-rank analysis of the probability of non-recurrence over time between treatment groups suggests no statistically significant difference between treatment groups (Figure S1, p=0.097). For the secondary outcome, 15/30 patients in the vancomycin group and 6/25 patients in the placebo group had positive cultures for VRE from stool (Table S1). Similar to the primary as randomized analysis, this difference in VRE carriage between groups achieves a p-value=0.048, and it is only marginally significant.

© 2025 Keating JA et al. *JAMA Network Open*.

eTable 1. As-Completed-Treatment Analysis of Primary and Secondary Outcomes				
	**Oral Vancomycin (Intervention) Group**	**Placebo (Control) Group**	**Absolute difference, % (2-sided 95% CI)**	**P-value**
	# of patients (%)	# of patients (%)		
**CDI recurrence within 8 weeks**[a]	15/31 (48.4)	19/29 (65.5)	18.1 (-41.8, 7.5)	0.18
**VRE carriage at 8 weeks**[a,b]	15/30 (50)	6/25 (24)	26 (1.5, 50.5)	0.048

CDI=*C. difficile* infection; VRE = vancomycin-resistant enterococcus

[a]The primary and secondary outcomes were analyzed using a Chi-squared test without continuity correction.

[b]Stool samples to measure VRE carriage were collected within ±14 days of 8 weeks following the end of treatment.

© 2025 Keating JA et al. *JAMA Network Open*.

eFigure 1. Estimated non-recurrence probability over time between treatment groups for the as-completed-treatment population.

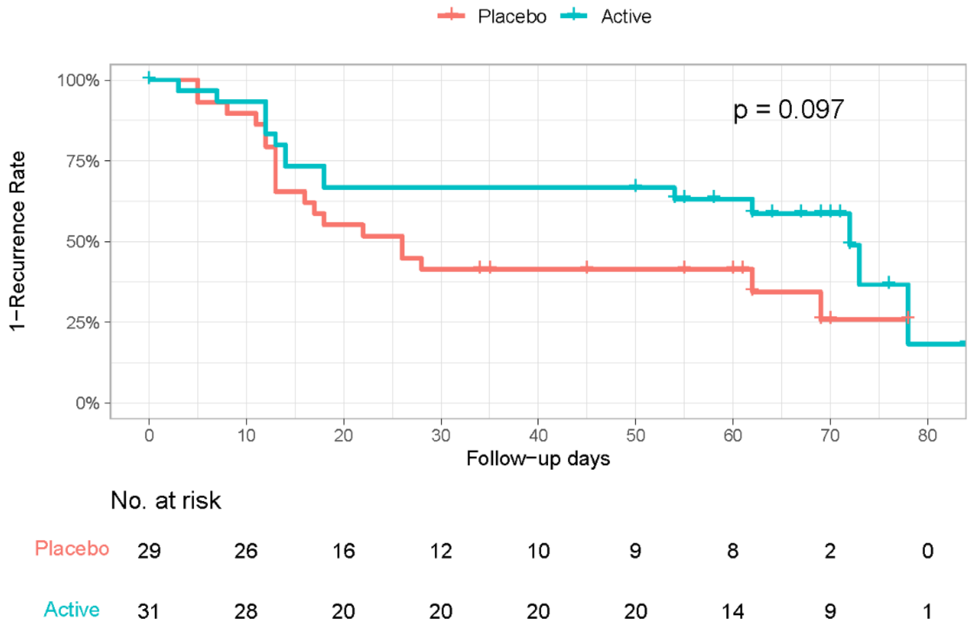

© 2025 Keating JA et al. *JAMA Network Open*.

eAppendix 2. Comparison of Primary Outcome, Stratified by Study Sites

We estimated the non-recurrence probability over time between intervention and control groups for the as randomized analysis, stratified by the study sites due to limited sample size (Figure S2). The overall curves were significantly different in the log-rank analysis ($p=0.041$). When comparing the recurrence rates between treatment groups stratified by study sites at 10-day intervals up to 80 days after completing the intervention, no statistically significant results were identified (Table S2).

© 2025 Keating JA et al. *JAMA Network Open*.

eFigure 2. Estimated non-recurrence probability over time between treatment groups for the as-randomized population, stratified by study sites.

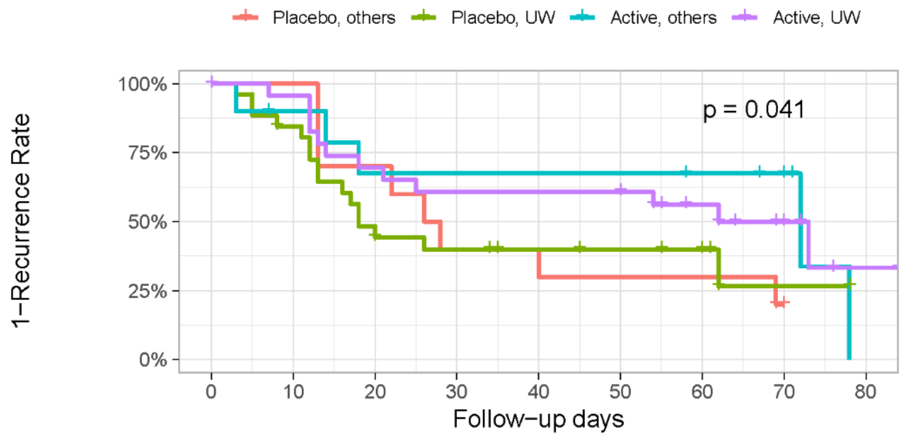

No. at risk

Placebo, others	14	10	7	4	4	3	3	1	0
Placebo, UW	28	21	12	9	7	6	5	1	0
Active, others	12	8	6	6	6	6	5	4	0
Active, UW	27	22	16	14	14	14	9	5	1

© 2025 Keating JA et al. *JAMA Network Open*.

**eTable 2. Comparison of recurrence rates between intervention group and placebo group at 10-day intervals after the intervention, stratified by study sites.**		
**Days**	**P-value (UW)**	**P-value (other sites)**
10	0.17	0.27
20	0.06	0.83
30	0.13	0.34
40	0.13	0.19
50	0.13	0.19
60	0.22	0.19
70	0.22	0.10
80	0.34	0.43

© 2025 Keating JA et al. *JAMA Network Open*.

eAppendix 3. Primary and Secondary Outcomes in Participants Taking Higher-Risk Antibiotics

Of 81 randomized participants, 78 participants had a record of the antibiotics being used and 73 participants used a higher-risk antibiotic. Higher risk antibiotics were defined as cephalosporins, fluoroquinolones, clindamycin, carbapenems, and beta-lactamase inhibitors.

© 2025 Keating JA et al. *JAMA Network Open*.

eTable 3. Primary and Secondary Outcomes in Participants Taking High-Risk Antibiotics

	Oral Vancomycin (Intervention) Group	Placebo (Control) Group	Absolute difference, % (2-sided 95% CI)	P-value
	# of patients (%)	# of patients (%)		
**CDI recurrence within 8 weeks[a]**	17/38 (44.7)	21/35 (60.0)	-15.3 (-37.9, 7.4)	0.1922
**VRE carriage at Visit 1[a,b]**	16/36 (44.4)	12/34 (35.3)	9.2 (-13.7, 32.0)	0.438
**VRE carriage at Visit 3[a,b]**	15/29 (51.7)	6/23 (26.1)	25.6 (0.09,51.2)	0.0613

CDI=*C. difficile* infection; VRE = vancomycin-resistant enterococcus
[a]The primary and secondary outcomes were analyzed using a Chi-squared test without continuity correction.
[b]Stool samples to measure VRE carriage were collected within ±14 days of Visit

© 2025 Keating JA et al. *JAMA Network Open*.

eFigure 3. Estimated non-recurrence probability over time between treatment groups for the randomized participants taking high-risk antibiotics.

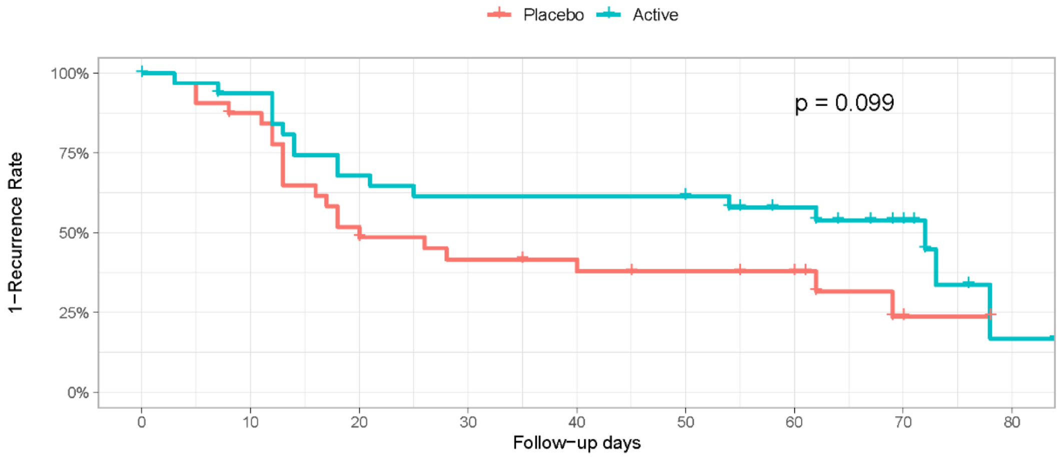

No. at risk

Placebo	35	27	16	12	11	9	8	2	0
Active	38	29	21	19	19	19	14	9	1

© 2025 Keating JA et al. *JAMA Network Open*.

eAppendix 4. Primary and Secondary Outcomes in Immunocompromised Participants

Of 81 randomized participants, 64 had a condition, disease, or surgery recorded in their medical history and 35 of these participants had a previous or current condition determined to be immunocompromising (e.g., cancer or transplant surgery).

© 2025 Keating JA et al. *JAMA Network Open*.

**eTable 4. Primary and Secondary Outcomes in Immunocompromised Participants**						
	**Oral Vancomycin (Intervention) Group**		**Placebo (Control) Group**		**Absolute difference, % (2-sided 95% CI)**	**P-value**
	# of patients (%)		# of patients (%)			
**CDI recurrence within 8 weeks[a]**	10/19 (52.6)		11/16 (68.8)		-16.1 (-48.1, 15.1)	0.3322
**VRE carriage at Visit 1[a,b]**	10/18 (55.6)		7/16 (43.8)		11.8 (-21.6, 45.2)	0.492
**VRE carriage at Visit 3[a,b]**	6/15 (40.0)		2/10 (20.0)		20 (-15.1, 55.1)	0.2936

CDI=*C. difficile* infection; VRE = vancomycin-resistant enterococcus
[a]The primary and secondary outcomes were analyzed using a Chi-squared test without continuity correction.
[b]Stool samples to measure VRE carriage were collected within ±14 days of Visit

© 2025 Keating JA et al. *JAMA Network Open*.

eFigure 4. Estimated non-recurrence probability over time between treatment groups for the randomized participants with an immunocompromising condition.

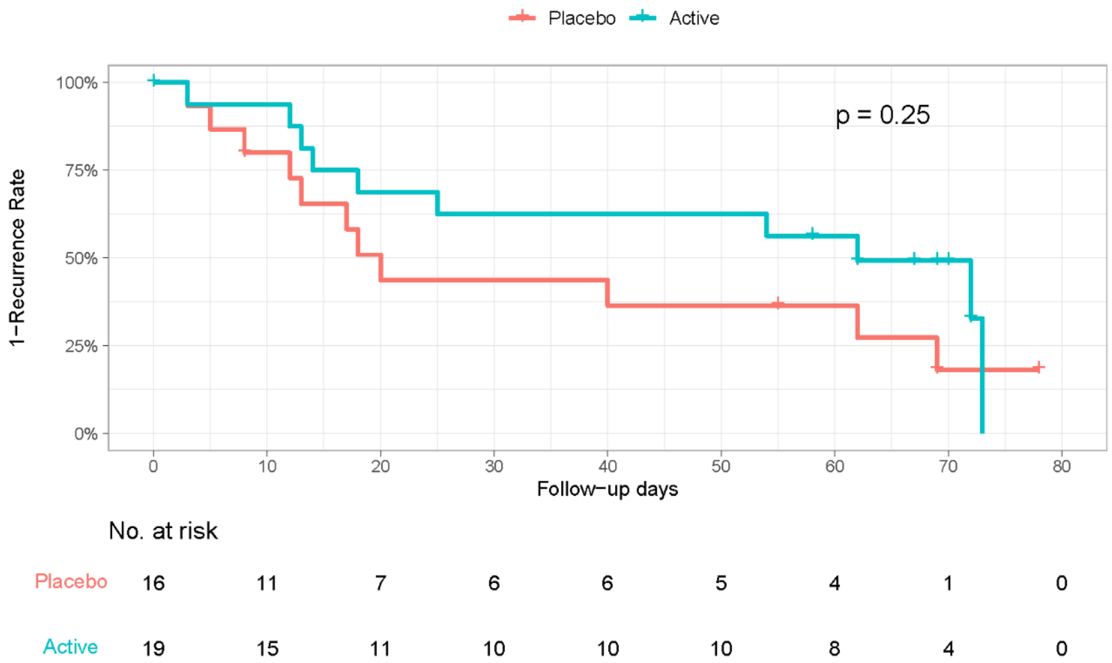

© 2025 Keating JA et al. *JAMA Network Open*.

eAppendix 5. Primary and Secondary Outcomes in Participants Aged 65 Years or Older

Of 81 randomized participants, 30 were aged 65 years or older.

© 2025 Keating JA et al. *JAMA Network Open*.

eTable 5. Primary and Secondary Outcomes in Participants 65 Years or Older				
	**Oral Vancomycin (Intervention) Group**	**Placebo (Control) Group**	**Absolute difference, % (2-sided 95% CI)**	**P-value**
	# of patients (%)	# of patients (%)		
**CDI recurrence within 8 weeks[a]**	4/14 (28.6)	8/16 (50.0)	-21.4% (-55.5, 12.6)	0.232
**VRE carriage at Visit 1[a,b]**	7/13 (53.8)	5/15 (33.3)	20.5% (-15.6, 56.6)	0.274
**VRE carriage at Visit 3[a,b]**	6/11 (54.5)	2/10 (20.0)	34.5% (-3.9, 73.0)	0.1035

CDI=*C. difficile* infection; VRE = vancomycin-resistant enterococcus

[a]The primary and secondary outcomes were analyzed using a Chi-squared test without continuity correction.

[b]Stool samples to measure VRE carriage were collected within ±14 days of Visit

© 2025 Keating JA et al. *JAMA Network Open*.

eFigure 5. Estimated non-recurrence probability over time between treatment groups for the randomized participants aged 65 years or older.

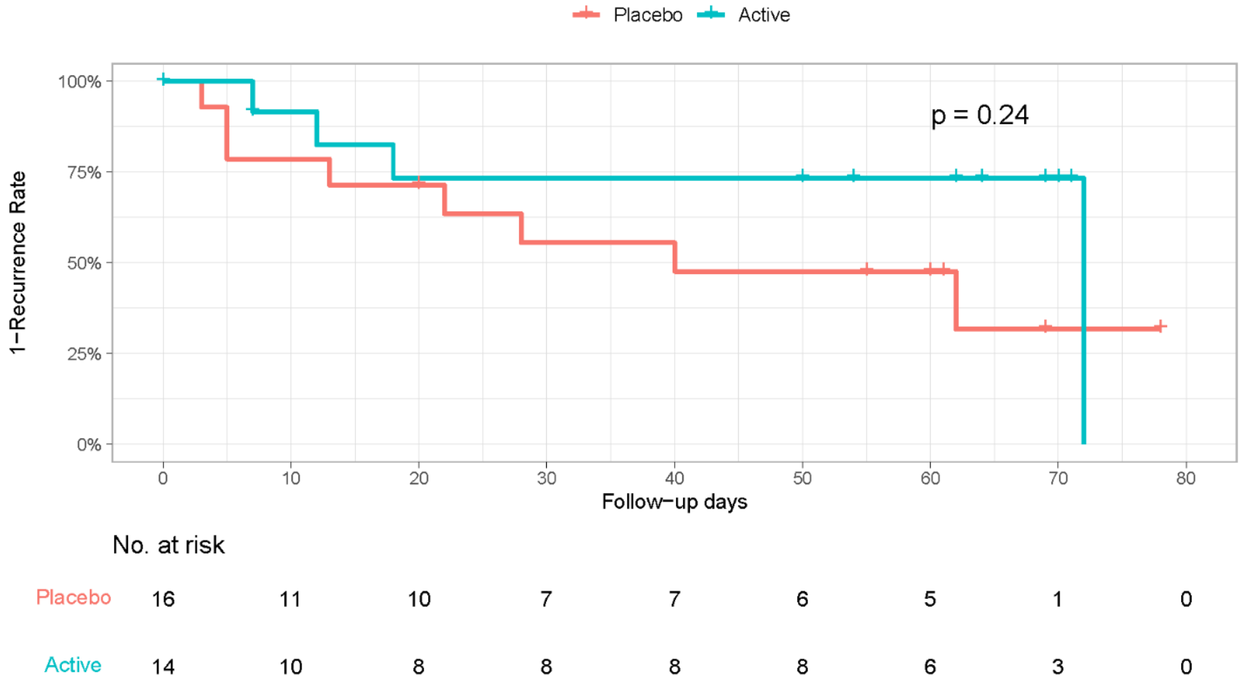

© 2025 Keating JA et al. *JAMA Network Open*.
