# Supplementary material for: Oral Vancomycin for Prevention of Recurrent Clostridioides difficile Infection: A Randomized Clinical Trial
Source: JAMA Netw Open. 2025 Jul 2;8(7):e2517834. doi: 10.1001/jamanetworkopen.2025.17834 (PMC12223870; doi:10.1001/jamanetworkopen.2025.17834)
Supplement: Supplement 3. — Data Sharing Statement [file jamanetwopen-e2517834-s003.pdf]

Data Sharing Statement

Keating. Oral Vancomycin for Prevention of Recurrent Clostridioides difficile Infection. *JAMA Netw Open*. Published July 02, 2025. doi:10.1001/jamanetworkopen.2025.17834

Data
Additional Information: ClinicalTrials.gov identifier: NCT03462459
https://clinicaltrials.gov/study/NCT03462459
Data available: Yes
Data types: Deidentified participant data
How to access data: De-identified data will be shared through publications and on ClinicalTrials.gov. Requests for data not made public through these mechanisms may require a data use and sharing agreement in accordance with AHRQ and University of Wisconsin-Madison data sharing policies; such requests should be made to the study Principal Investigator Nasia Safdar (ns2@medicine.wisc.edu).
When available: With publication

Supporting Documents
Document types: None

Additional Information
Who can access the data: Researchers whose proposed use of the data has been approved.
Types of analyses: Any purpose.
Mechanisms of data availability: With investigator support and fully executed data use agreement.
Any additional restrictions: Local IRB approval will be required for any data sharing.
